# HMGB1/RAGE axis mediates stress-induced RVLM neuroinflammation in mice via impairing mitophagy flux in microglia

**DOI:** 10.1186/s12974-019-1673-3

**Published:** 2020-01-10

**Authors:** Shutian Zhang, Li Hu, Jialun Jiang, Hongji Li, Qin Wu, Kokwin Ooi, Jijiang Wang, Yi Feng, Danian Zhu, Chunmei Xia

**Affiliations:** 1 Department of Physiology and Pathophysiology, School of Basic Medical Sciences, Fudan University, No. 130, Dongan Road, Shanghai, 200032 People’s Republic of China; 2 Clinical Medicine (Eight-year Program), Shanghai Medical College, Fudan University, Shanghai, 200032 People’s Republic of China; 3 Laboratory of Neuropharmacology and Neurotoxicology, Shanghai Key Laboratory of Bio-Energy Crops, College of Life Science, Shanghai University, Shanghai, 200444 People’s Republic of China; 4 Department of Integrative Medicine and Neurobiology, School of Basic Medical Sciences, Fudan University, Shanghai, 200032 People’s Republic of China

**Keywords:** Microglia, Stress, Hypertension, Mitochondria, High-mobility group box 1, RAGE, Autophagy, Neuroinflammation

## Abstract

**Background:**

Microglial mediated neuroinflammation in the rostral ventrolateral medulla (RVLM) plays roles in the etiology of stress-induced hypertension (SIH). It was reported that autophagy influenced inflammation via immunophenotypic switching of microglia. High-mobility group box 1 (HMGB1) acts as a regulator of autophagy and initiates the production of proinflammatory cytokines (PICs), but the underlying mechanisms remain unclear.

**Methods:**

The stressed mice were subjected to intermittent electric foot shocks plus noises administered for 2 h twice daily for 15 consecutive days. In mice, blood pressure (BP) and renal sympathetic nerve activity (RSNA) were monitored by noninvasive tail-cuff method and platinum-iridium electrodes placed respectively. Microinjection of siRNA-HMGB1 (siHMGB1) into the RVLM of mice to study the effect of HMGB1 on microglia M1 activation was done. mRFP-GFP-tandem fluorescent LC3 (tf-LC3) vectors were transfected into the RVLM to evaluate the process of autolysosome formation/autophagy flux. The expression of RAB7, lysosomal-associated membrane protein 1 (LAMP1), and lysosomal pH change were used to evaluate lysosomal function in microglia. Mitophagy was identified by transmission electron microscopic observation or by checking LC3 and MitoTracker colocalization under a confocal microscope.

**Results:**

We showed chronic stress increased cytoplasmic translocations of HMGB1 and upregulation of its receptor RAGE expression in microglia. The mitochondria injury, oxidative stress, and M1 polarization were attenuated in the RVLM of stressed Cre-CX3CR1/RAGE^fl/fl^ mice. The HMGB1/RAGE axis increased at the early stage of stress-induced mitophagy flux while impairing the late stages of mitophagy flux in microglia, as revealed by decreased GFP fluorescence quenching of GFP-RFP-LC3-II puncta and decreased colocalization of lysosomes with mitochondria. The expressions of RAB7 and LAMP1 were decreased in the stressed microglia, while knockout of RAGE reversed these effects and caused an increase in acidity of lysosomes. siHMGB1 in the RVLM resulted in BP lowering and RSNA decreasing in SIH mice. When the autophagy inducer, rapamycin, is used to facilitate the mitophagy flux, this treatment results in attenuated NF-κB activation and reduced PIC release in exogenous disulfide HMGB1 (ds-HMGB1)-stimulated microglia.

**Conclusions:**

Collectively, we demonstrated that inhibition of the HMGB1/RAGE axis activation led to increased stress-induced mitophagy flux, hence reducing the activity of microglia-mediated neuroinflammation and consequently reduced the sympathetic vasoconstriction drive in the RVLM.

**Electronic supplementary material:**

The online version of this article (10.1186/s12974-019-1673-3) contains supplementary material, which is available to authorized users.

## Introduction

The etiology of hypertension is extremely complex, involving both a strong genetic predisposition and multifactorial in pathogeneses. Nowadays, globalization, cultural communications, socioeconomic development, and professional competition agitate more and more people to suffer chronic psychosocial stress, anxiety, and depression. Data showed that psychosocial stress was associated with an increased risk of hypertension [1]. Acute stressor exposure for 24 h elevates basal sympathetic nervous system (SNS) tone and exaggerates SNS responses in experimental animals exposed to the subsequent repeated stressors, which were indicated by decreased heart rate variability and elevated peripheral noradrenaline [2, 3].

It has been demonstrated that the augmenting sympathetic activity underlies the pathogenesis in hypertension and other cardiovascular events [4]. Humans with borderline hypertension and/or prehypertensive rat models show an elevation in centrally mediated sympathetic activity and an augmented neuroinflammatory state [5]. Overactive brain local renin-angiotensin system (RAS), oxidative stress, and neuroinflammation in the brainstem cardioregulatory centers rostral ventrolateral medulla (RVLM) and other brain regions such as hypothalamic paraventricular nucleus (PVN) are the key factors in augmenting sympathetic activity [6, 7]. Furthermore, it has been well established that sympathetic excitation and increased blood pressure (BP) are resulted from elevated oxidative stress and inflammation in the PVN, which excite PVN neurons [8].

In hypertension, angiotensin (Ang) II, one of the main components of RAS, plays the roles of triggering proinflammatory cytokine (PIC) release and reactive oxygen species (ROS) production both in microglial and astroglial cells [9]. Upon activation, resting microglia (MG) can be switched into proinflammatory/classic (M1) or anti-inflammatory/alternative (M2) immunophenotypes [10]. We have previously reported that the activated MG are increased in the RVLM of stress-induced hypertension (SIH) rats, and the secretion of TNF-α, IL-1β, and other PICs by microglia-mediated response resulted in increased BP [11]. However, the mechanisms by which stress triggers the activation of MG remain unclear.

High-mobility group box 1 (HMGB1) functions as an alarmin protein or damage-associated molecular pattern (DAMP) in response to neuroinflammation, and it is taken as the alarmin that mediates activation of the innate immune response including the release of chemotactic factors and PICs [12]. It was shown that the stress hormone such as glucocorticoids reached a critical threshold after animals had been exposed to acute and/or chronic stressors. Stressor setting thereby induces synthesis and release of HMGB1 from microglia which triggers the innate immune system (e.g., NLRP3 inflammasome priming) for sensitizing the proinflammatory response of microglia to subsequent immune challenges such as injury and depression, especially during a fight/flight response [13, 14]. HMGB1 plays essential roles in the regulation of autophagy in intranuclear, cytosolic, and extracellular compartment, e.g., advanced glycation end product receptor (RAGE) of HMGB1 controls myocardial dysfunction and oxidative stress in high-fat-fed mice by sustaining the mitochondrial dynamics and autophagy-lysosome pathway [15]. However, the detailed mechanisms by which stressors prime microglia-mediated neuroinflammation remain unclear.

In the preliminary experiment, we found that rats and/or mice subjected to electric foot shock with noise stressor showed injured and giant mitochondria in both neurons and microglia in the RVLM. Mitochondrial fusion, fission biogenesis, and mitophagy profiles determine the dynamics of mitochondrial turnover. Mitophagy is a selective form of autophagy, which is important in maintaining mitochondrial homeostasis [16]. Once the injured mitochondria are engulfed by phagophore, mitophagy proceed to autophagic flux, which features production and lysosomal degradation of autophagosomes constantly and cargos therein [17, 18]. It was shown that defective mitophagy flux resulted in accumulation of damaged mitochondria and finally led to various neurodegenerative diseases, including Alzheimer’s disease (AD) [19]. In neurons, we found that the mitophagy flux was blocked in the RVLM of SIH rats [11]. This spurs our interest to investigate how mitophagy flux affect microglia-mediated neuroinflammation in the RVLM of SIH mice.

It was evidenced that mitochondrial dysfunction (including mitophagy) and neuroinflammation interacts as a bidirectional causal relationship [20]. Here, the mechanisms of HMGB1 as a potential mediator in priming stress-induced microglia were explored. Our present study confirmed that HMGB1/RAGE axis mediates impairment of stress-induced mitophagy flux; consequently, the activated microglia undergo immunophenotypic switching, thus leading to neuroinflammatory response such as NF-κB activation and PIC release. In particular, priming of the microglia-mediated neuroinflammatory response further induces pathogenesis of SIH. Crucially, HMGB1/RAGE axis might be a novel target for the prevention and treatment of stress-related hypertension.

## Materials and methods

### Drugs and reagents

Lyophilized ds-HMGB1 was obtained from HMGBiotech (Milan, IT). Artificial cerebrospinal fluid (aCSF) was purchased from Leagene (CZ0530, Leagene Biotecnology, Beijing, China). Tamoxifen (T5642), rapamycin (V900930), and chloroquine (C6628) were purchased from Sigma-Aldrich (Sigma, St Louis, MO, USA). AAV9-mRFP-GFP-LC3/GFP-LC3 was purchased from Hanbio Co. Ltd. (Shanghai China). LV-LoxP-stop-LoxP-shRNA-RAGE, the HMGB1 siRNA plasmids, and negative control plasmids were purchased from Shanghai Genechem Co. Ltd. (Shanghai, China). Lyso-Tracker Red DND-99 (L7528) and Lyso-Sensor Green DND-189 (L7535) were purchased from Invitrogen (Carlsbad, CA, USA). DAPI (C1002, Beyotime, Shanghai, China), MitoTracker Green (C1048), and MitoTracker Red (C1049) were purchased from Beyotime (Shanghai, China). Seahorse XFp Cell Energy Phenotype Test Kit (103275-100) was purchased from Agilent Technologies (Palo Alto, CA, USA). Pierce Protein A/G Agarose (20421) was provided by Thermo Fisher Scientific (Waltham, USA). The flowing antibodies: HMGB1 (ab79823); OX42 (ab48004,); β-actin (ab179467); Histone (ab1791); Acetylated lysine (ab193); CD86 (ab119857); TLR2 (ab209217); TLR4 (ab22048); TLR9 (ab134368); RAGE (ab3611); IL-1β (ab9722); LC-3 I/II (ab128025); p62 (ab109012); PINK1 (ab23707); PRKN (ab77924); ATG5 (ab108327); BECN1 (ab210498); p-p65 (ab86299); Rab7 (ab137029); LAMP1 (ab24170); c-fos (ab156802); PGP9.5 (ab108986); Donkey Anti-Goat IgG H&L (Alexa Fluor 488) (ab150129); Donkey Anti-Goat IgG H&L (Cy5) (ab6566); Donkey Anti-Rabbit IgG H&L (Alexa Fluor 647) (ab150075); Donkey Anti-Rabbit IgG H&L (Alexa Fluor 488) (ab150073) and Donkey Anti-Rabbit IgG H&L (HRP) (ab6802), were purchased from Abcam (Cambridge, UK). The Mouse Noradrenaline (NE) ELISA Assay kit (NOU39-K010) was provided by Eagle Biosciences (Amherst, NH).

### Experiment design

- Experiment 1: To explore the roles and expression of HMGB1/RAGE axis in stress-induced hypertension in vivo, the C57BL/6 wild type (WT) male mice weighing 25 ± 5 g were randomly assigned to one of three groups: (1) control group (Ctrl), (2) stress group, (3) stress + HMGB1 silencing group (stress + siHMGB1), and (4) stress + microglia-specific deletion RAGE group (Cre-CX3CR1/RAGE ^fl/fl^) mice were also included. The mice in stress + siHMGB1 mice received siRNA microinjections into the RVLM. The effects of RNA interference on the HMGB1 were tested after administration of HMGB1 siRNA or control siRNA (siHMGB1 or siCtrl) into the RVLM for 2 weeks in stressed mice (Additional file 1: Figure S1).
- Experiment 2: To investigate the roles of HMGB1/RAGE axis in microglia activation as well as to identify the contribution of microglia-derived HMGB1 to stress response, the microglia were obtained and cultured ex vivo. The microglia in each cohort were obtained from one of the abovementioned four groups.
- Experiment 3: It has been confirmed that the proinflammatory disulfide HMGB1 (dsHMGB1) in microglia induces the expression of NLRP3 and dsHMGB1 is involved in the microglia-mediated neuroinflammation [13, 14]. To examine the effects of HMGB1 on mitophagy flux and proinflammatory cytokine release, the microglia were treated with dsHMGB1 in vitro. The primary cultured microglia were assigned into a (1) vehicle control group (Ctrl), (2) dsHMGB1 group, (3) dsHMGB1 + chloroquine (lysosomal inhibitor) group, and (4) dsHMGB1+ rapamycin (autophagy inducer) group[21, 22].

For the abovementioned experiments, each cohort (*n* = 6 per group) was used to perform immunofluorescence, immunoblot, qPCR, BP measurement, and/or other experiment analysis.

### Animal preparation

The C57BL/6 wild type (WT) male mice were used in our present study and were obtained from Animal Laboratory Center of Fudan University. Altogether, 152 animals were used in this study, which include a mortality rate of 1% (mice with intolerance for brain surgery, or death during stress). The mice were housed in a 12-h light/dark cycle, temperature-controlled room with standard 24 °C and with food and tap water ad libitum. All efforts were made to minimize the number of animals used and their suffering.

The design of the SIH model of mice was modified based on previous publications [11, 23, 24]. In short, mice were subjected to electric foot shock with noises. They were put in a Plexiglas chamber (26 cm × 21 cm × 26 cm) with a grid floor made of stainless-steel rods (0.3 cm diameter, spaced 1.0 cm apart), connected to a shock generator. A series of foot shocks of 0.5-mA intensity of 1-s duration with a shock interval of 4 min were controlled electronically. Synchronously, noises (range 88–98 dB) produced by a buzzer were given as a conditioned stimulus. After acclimatization for a few times, the stressed group mice were subjected to stress for 2 h twice daily for 15 consecutive days. The control group underwent sham stress.

During the days of stress, the BP of mice was recorded using the noninvasive tail-cuff method (CODA; Kent Scientific, Torrington, Connecticut, USA). Prior to BP measurements, the animals were placed into the restraining chambers on a warm platform for 30 min to ensure adaptation to the procedure. BP was recorded in a proper environment (RT, lightning, and noise-free atmosphere). BP measurements were repeated three times, and the average value was taken.

### Generation of mice with RAGE deficiency in microglia of the RVLM (Cre-CX3CR1/RAGE ^fl/fl^ mice)

CX3CR1^CreER^ mice were purchased from the Jackson Laboratory (020940, Jackson Lab). For the generation of Cre-CX3CR1/ RAGE ^fl/fl^ mice, the gene transfer of LV-LoxP-stop-LoxP-shRNA-RAGE was performed by microinjection bilaterally into the RVLM of CX3CR1^CreER^ mice 14 days prior to tamoxifen administered as was mentioned in a previous study [25, 26]. Briefly, the siRNA virus was injected bilaterally into the RVLM of mice employing a mouse stereotaxic instrument under isoflurane anesthesia (~ 2%). The animals respired room air spontaneously via tracheas intubated with noninvasive polyethylene tubes. Heads of the mice were affixed on a stereotaxic apparatus (Neurostar Tubingen, Germany) and flexed to an angle of approximately 45°. The occipital bone was carefully removed to expose the fourth ventricle, the floor of which was kept horizontally. A glass micropipette was inserted into the RVLM (1.2 mm lateral to the midline, 5.3 mm ventral to the dorsal surface of the brain, 1.9 mm caudal to lambda) according to the atlas of Paxinos and Franklin [27]. Following the microinjection, the muscle layers covering the fourth ventricle were sutured. The mice’s body temperature was maintained at 37 °C with heating pads until the animals recovered from surgery. The microinjection site was identified by neutral red staining after the mice were sacrificed (see Additional file 2). The total microinjection volumes were 0.1 μl which was consistent with our previous study [11]. The siRNA was transferred into the RVLM bilaterally and administered into each injection site over 10–15 min by pressure through a glass micropipette.

To delete the RAGE gene targeting the RVLM microglia, tamoxifen was dissolved in warm corn oil and administered subcutaneously twice 48 h apart. The efficiency of RAGE knockout was tested using immunoblot, RT-PCR, and RAGE immunofluorescent staining of the RVLM tissue, respectively (see Additional file 3).

### Microglia isolations and ex vivo treatments

The RVLM contains both neurons and different types of glia, in order to study and examine the effects of vivo treatments on microglia; isolation of microglia from CNS tissue was commonly used. Isolation of microglia from regional brain specifically identifies microglia immunophenotype and function comparing with that of whole-brain microglia. Frank et al. [28] report the detailed procedure for the rapid isolation of microglia from discrete CNS anatomical loci. In addition, they confirm that the isolation microglia procedure preserves the in vivo phenotype of microglia in situ. Hence, our ex vivo experiment was dependent on their experimental method with minimal procedural confounds for investigating in vivo treatments [28].

The RVLM tissue were identified as a previous atlas described [47], and RVLM microglia were isolated using a Percoll density gradient as Frank et al. described [28]. The immunophenotype and purity of microglia were confirmed using immunohistochemistry staining of a microglia marker (CD86) (see Additional file 4). Briefly, mice brains were rapidly extracted, and the RVLM was dissected out on ice. The RVLM was homogenized in 3 mL of 0.2% glucose in 1× DPBS in a sterilized glass homogenizer and the homogenate was strained through a 40-μm filter (Falcon) that was rinsed with an additional 2 mL or DPBS. The homogenate was transferred to a sterile 5-mL tube and pelleted at 1000*g* for 10 min at 22 °C. Supernatant was aspirated off, and a Percoll gradient was created by resuspending the pellet in 2 mL of 70% isotonic percoll, followed by a layer of 2 mL 50% Percoll and topped with 1 mL DPBS. The gradient was spun at 1200*g* for 45 min at 22 °C with no acceleration or break. Myelin debris was removed, and then microglia were extracted from the 50/70% interface. Microglia were then washed in DPBS and pelleted at 1000*g* for 10 min at 22 °C. Following isolations, microglia were resuspended in media (sterile high glucose DMEM + 10%FBS) that was passed through a 0.2-μm filter. Microglia concentration was adjusted to a density of 8000 cells/100 μL, and cells were plated in a 96-well v-bottom plate.

### Adenovirus transfection

The AAV9-mRFP-GFP-LC3 transfection was described as our previous publication and as the manufacturer’s instructions [11]. In brief, mRFP-GFP-LC3 was delivered via adeno-associated virus (AAV) (Hanbio, Shanghai, China). The GFP signals were quenched in the lysosomal acidic conditions, whereas mRFP fluorescence was relatively stable. Therefore, this tandem-tagged fluorescent protein showed yellow puncta in the autophagosomes, while exhibited red only in lysosomes [29]. Quantification of GFP and mRFP fluorescence puncta, and colocalization between two different signals were recorded and analyzed using Fiji ImageJ software. The microinjection site was identified by neutral red staining after the mice were sacrificed.

### Renal sympathetic nerve activity (RSNA) recording

To summarize, the mice were anesthetized as the abovementioned method, with a mixture of urethane and chloralose in an intraperitoneal dose of 7 ml/kg. In the retroperitoneal space through a left flank incision on mouse, a bundle of left renal sympathetic nerves was isolated, and a pair of platinum-iridium electrodes was placed on them. The nerve-electrode complex was covered with silicone gel (Kwik-Sil, WPI, Sarasota, FL). The RSNA signal was amplified (× 1000) and filtered (bandwidth 30–3000 Hz) using a Grass P55C preamplifier, and then was input into a PowerLab (AD Instruments, Australia) data-acquisition system, from which the signal was monitored, recorded, and saved in a computer using the LabChart 7 software.

### Flow cytometric analysis of M1 phenotype

To measure M1 polarization, primary isolation and culture of mouse microglial cells were carried out as described in our previous study [30]. Microglia were harvested by resuspending in cold PBS containing 0.5% BSA/0.05% NaN_3_, then incubating in 20% DMEM/F12 medium. Microglia were surface-stained with PE-conjugated anti-CD86 with fixation and permeabilization buffer as per the manufacturer’s instructions. Microglia surface staining was then assessed by flow cytometry (FACS Calibur running CellQuest Pro; Becton Dickinson, UK) and analyzed using Flowing software v2.5.1.

### Transmission electron microscopy

The procedures were reported as in our previous publication [11, 30]. In brief, for electron microscopy (EM) embedding, the cell medium was decanted, and Karnovsky’s fixative (2% paraformaldehyde plus 2.5% glutaraldehyde in 0.1 m phosphate buffer, pH 7.2–7.4) was added to a depth of about 5 mm. Cells were then fixed, osmicated, rinsed with phosphate buffer, dehydrated, and embedded in epoxy resin, which was allowed to polymerize for 24 h at 70 °C. Blocks containing microglia were sectioned using an ultramicrotome (Ultracut; Leica) at 70–80 nm. Thin sections were collected on grids and stained with uranyl acetate and lead citrate. Grids were examined under a transmission electron microscope (H-700; Hitachi, Tokyo, Japan) at 80 kV.

### Double immunofluorescence staining, imaging, and analysis

The mouse was subjected to anesthesia as mentioned above, then the left ventricle was perfused with 200 ml of 0.01 M PBS, (pH 7.4), followed by 200 ml of freshly prepared 4% paraformaldehyde in 0.1 M PB, respectively. The RVLM sections were collected and removed to post-fixation for 4 h, then they were placed in 20% and 30% sucrose at 4 °C to dehydration overnight, respectively. Free-floating 30-μm coronal sections containing the RVLM were cut using a cryostat (Microm, Germany). The coronal sections of the RVLM were washed in PBS and then incubated with 0.3% Triton X-100 for 30 min followed incubation by 5% horse serum for 1 h at 37 °C to block non-specific protein. The sections were incubated with rabbit polyclonal antibody to HMGB1, RAGE, CD86, c-fos, OX42 (a microglia marker), and/or PGP9.5 (a neuron marker) for overnight at 4 °C. The Donkey Anti-Goat IgG H&L (Alexa Fluor 488), Donkey Anti-Goat IgG H&L (Cy5), Donkey Anti-Rabbit IgG H&L (Alexa Fluor 647), and Donkey Anti-Rabbit IgG H&L (Alexa Fluor 488) secondary antiserum were used as secondary antibody. LC-3 colocalization with MitoTracker was investigated in vitro. The fluorescent signal was monitored under a Fluorview FV300 laser scanning confocal microscope (Olympus, Tokyo, Japan); immunoreactivity manifested specific green or red fluorescence.

### Immunofluorescent imaging and analysis

These images were processed and filtered using ImageJ software in order to bring out the foci of the pictures. These foci were then automatically segmented by thresholding, and pixel-by-pixel colocalization analysis of the segmented points from the two channels was assessed using the ImageJ plugin Just Another Colocalization Plugin (JACoP), which calculated Pearson coefficients, indicating the percentage of thresholded pixels in the green channel that were occupied by corresponding thresholded pixels in the red channel. For statistical analysis of channel overlap data, ANOVA analysis was carried out using JMP 12 software [35].

### Western blot analysis, immunoprecipitation and quantitative real-time polymerase chain reaction (PCR)

Microglia isolated from the RVLM tissue of each mouse were homogenized in lysis buffer with 1% NP40, 1 mM PMSF. In brief, protein samples (20 μg each) were subjected to SDS/PAGE in 8–12% gradient gel (Invitrogen, Carlsbad, CA, USA) and were transferred to PVDF membrane. HMGB1 (1:1000), acetylated lysine (1:1000), TLR2 (1:1000), TLR4 (1:1000), TLR9 (1:1000), RAGE (1:1000), LC-3 I/II (1:1000), p62 (1:1000), PINK1 (1:1000), PRKN (1:1000), ATG5 (1:1000), BECN1 (1:1000), p-p65 (1:1000), Rab7 (1:1000), and LAMP1 (1:1000) were measured. This was followed by incubation with horseradish peroxidase-conjugated Donkey Anti-Rabbit IgG. The amount of detected protein was assessed using ECL detection reagents (WBKLS0050; Millipore), and the immunostaining band was visualized and quantitated by a fully automatic chemiluminescence image analysis system (Tanon-5200; Tanon Science & Technology, Shanghai, China). β-actin (1:5000) was developed as a loading control to normalize the data. In immunoprecipitation, cell lysates were pre-cleared with 1 μg normal rabbit IgG and 20 μl protein A+G Agarose beads for 2 h at 4 °C. After centrifugation at 1000×*g* for 5 min, supernatants were transferred to new tubes and incubated with 40 μl protein A+G Agarose beads and rabbit anti-HMGB1 antibody overnight at 4 °C. Beads were collected for immunoblotting after centrifugation and washed three times with PBS. Both nuclear and cytoplasm expression protein (HMGB1 and p65) were measured using a nuclear-cytoplasm separation method. The nucleus-cytoplasm separation assay was performed using the Nucleus-cytoplasm Protein Extraction Kit (Beyotime Biotechnology, Shanghai, China). Extracted nuclear and cytoplasmic proteins were resolved by SDS-PAGE and then immunoblotted with the indicated antibodies.

The total RNA extraction reagent (TaKaRa, Dalian, China) was used to extract the total RNA from the RVLM. The mRNAs of TLR2, TLR4, TLR9, RAGE, HMGB1, IL-1β, and TNF-α were analyzed by quantitative real-time PCR. Isolated mRNA was quantified by spectrophotometry, and the optical density 260/280 nm ratio was determined. The cDNA was synthesized using a high-capacity cDNA reverse transcription kit (Applied Biosystems, ABI). The relative quantification of gene expression was expressed as fold changes via normalization against β-actin by using the 2DDCT method. Primers used in this study were synthesized and provided by Majorbio Bio-Pharm Technology (Shanghai, China). The sequences of primers were designed using Primer Express 2.0 and are listed in Table 1.

**Table 1:** Sense and antisense primer sequences in quantitative RT-PCR

Gene name	Primer sequence	
	Forward	Reverse
TLR2	5′-GCTCCTGCGAACTCCTATC-3′	5′-CAGCAGACTCCAGACACCA-3′
TLR4	5′-CCTGACACCAGGAAGCTTGAA-3′	5′-TCTGATCCATGCATTGGTAGGT-3′
TLR9	5′-GCCTCCGAGACAACTACCTA-3′	5′-CTGCTGACATCCAGTTTCTG-3′
RAGE	5′-ACATGTGTGTCT-GAGGGAAGC-3′	5′-AGCTCTGACCGCAGTGTAAAG-3′
IL-1β	5′-ATGTGCTGCTGCGAGATTTG-3′	5′-CTCAACTGTGAAATGCCACC-3′
TNF-α	5′-GGAAAGCATGATCCGAGATG-3′	5′-CAGTAGACAGAAGAGCGTGGTG-3′
HMGB1	5′-GGCGAGCATCCTGGCTTATC-3’	5′-GGCTGCTTGTCATCTGCTG-3′
β-actin	5′-TTGCTGACAGGATGCAGAAGGAG-3′	5′-ACTCCTGCTTGCTGATCCACATC-3′

### ELISA

Serum norepinephrine from different groups of mice was assayed by using ELISA kits according to the manufacturer’s instructions.

### Lysosomal pH measurement

To measure lysosomal pH, cells were loaded with 0.5 μM Lyso-Tracker Red DND-99 in regular medium for 1 h at 37 °C in the dark. After being washed with PBS three times, cells were immediately observed under a fluorescent microscope. Lysosomal pH quantification was further performed using the lysosomal pH probe Lyso-Sensor Green DND-189. Briefly, cells were loaded with 1 mg/ml Lyso-Sensor overnight. Cells were then washed, trypsinized, and resuspended in PBS. The fluorescences were monitored using the Confocal laser scanning microscope. Lower 488/590 ratio indicate less acidic lysosomal pH.

### Measurement of mitochondrial oxygen consumption rate

The oxygen consumption rate (OCR) was measured by the extracellular flux analyzer XF24 (Agilent Seahorse XFe96, Santa Clara, CA, USA). Microglia of different groups were plated at 4 × 10^5^ cells/well in a Seahorse 24-well V7 microplate and cultured in complete DMEM growth medium for 24 h in a 5% CO_2_ incubator at 37 °C. Then, the medium was removed, and cells were incubated in XF assay medium in the absence of NaHCO_3_ and FBS for 1 h at 37 °C in a measuring chamber without CO_2_ input. The mitochondrial complex inhibitors (oligomycin, FCCP, and rotenone) were freshly prepared in XF assay media. After 32 min of measuring the basal respiration, oligomycin (2.5 μM) was injected into each well at 50 min, followed by FCCP (1 μM) at 74 min and rotenone (2.5 μM) at 98 min. OCR was recorded as pMoles per minute, and calculated as percentage of the OCR value before the treatment of tested agents. ATP turnover and respiratory capacity were measured and calculated after the sequential treatments with oligomycin and FCCP as previously described [47]. Averages of three wells were taken per data point. FCCP is an uncoupling agent of electron transport and can generate a proton efflux to induce the maximum respiration termed as respiratory capacity or uncoupled respiration.

### Mitochondrial ROS measurement

Mitochondrial ROS were measured following the manufacturer’s instructions of MitoSOX Red kit (Thermo Fisher Scientific). Briefly, treated cells cultured on glass were washed and incubated with 0.5 μM MitoSOX. After reaction, cells were washed and observed by an inverted fluorescence microscope for detection of superoxide anion (red fluorescence). Samples without MitoSOX Red Reagent were used to subtract the background. Mean fluorescence intensity was determined, and all samples were normalized to control samples.

### Collection of cerebrospinal fluid (CSF)

We followed the protocol provided by Lim NK et al. [31], which was presented in video form regarding procedures to collect cerebrospinal fluid from anesthetized mice. They provided a technique for drawing the abundant (an average of 10–15 μl) CSF from anesthetized mice. This improved method was in line with a currently known method of CSF collection to minimize contamination from blood.

### Statistical analysis

Experimental data were expressed as mean ± SEM (standard error of mean). Student’s unpaired *t* test was used for experiments that contained two groups of samples. For comparison of multiple groups, one-way or two-way analysis of variance with repeated measures was used to determine differences between groups. When an ANOVA was significant, post hoc testing of differences between groups was performed using the least significant difference (LSD) test. *P* < 0.05 indicated that the differences were statistically significant. Statistical data was analyzed with GraphPad Prism 5 software.

## Results

### Stress-induced HMGB1 upregulation and its cytoplasmic translocation in microglia of the RVLM

Based on previous reports, HMGB1 plays a critical role in the immune response via the translocation from the nucleus to the cytoplasm, and intracellular environment to the extracellular environment [32]. Here, the HMGB1 expression in the RVLM, microglia, and cerebrospinal fluid (CSF) was assessed using immunofluorescent, immunoblot, and enzyme-linked immunosorbent assay (ELISA), respectively.

**Fig. 1:**
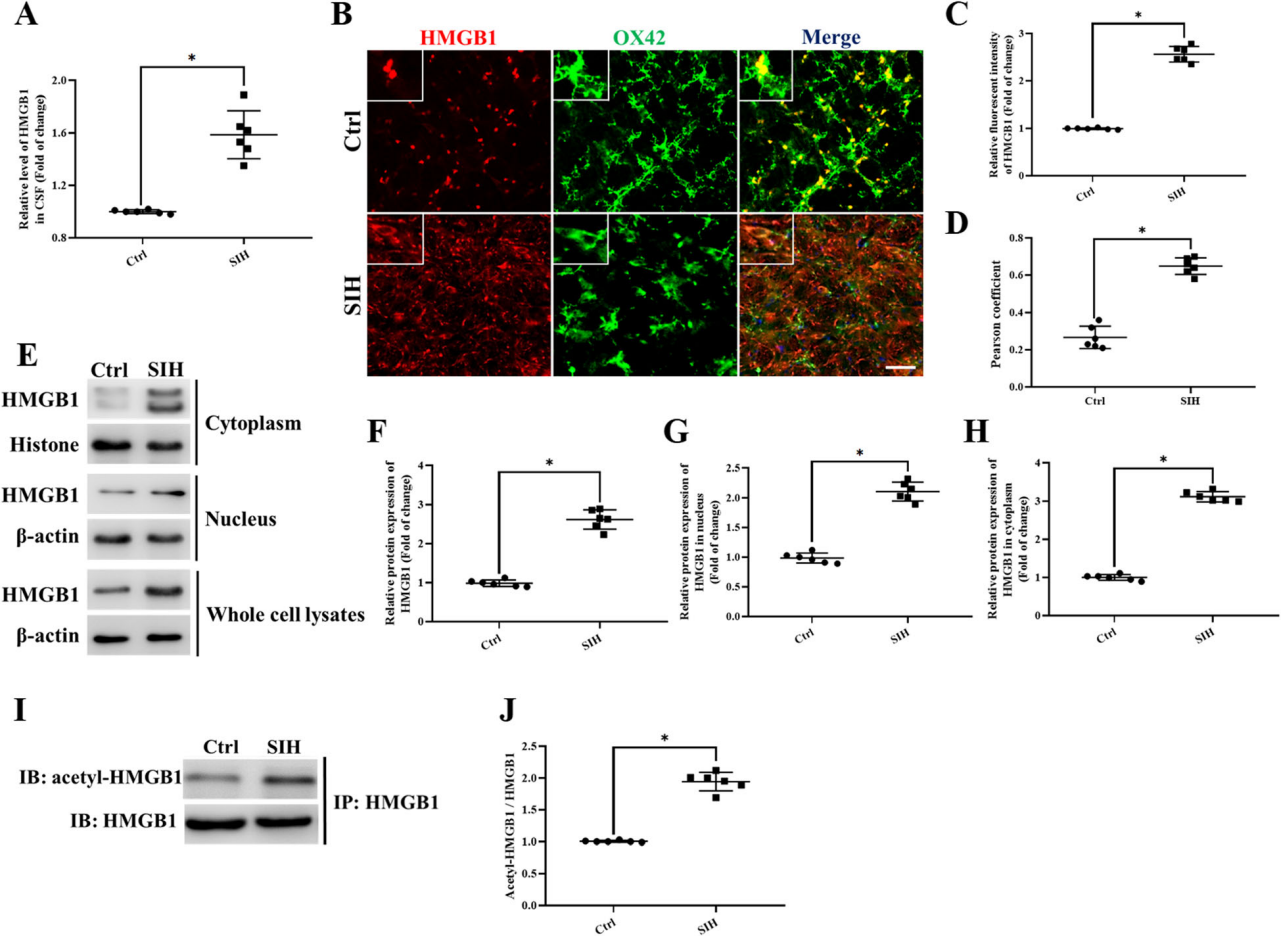
The levels of the HMGB1 protein both in CSF and in the RVLM was upregulated after 15 days of stress exposure in mice. **a** ELISA analysis showed the HMGB1 level in CSF. **b** Double immunofluorescent staining of HMGB1 in the RVLM of mice brain. The colocalization of HMGB1 with microglia indicates HMGB1 expression both in cytoplasm and nuclear in microglia (red, HMGB1; green, microglia; blue, nucleus/DAPI) (scale bar = 30 μm). **c** Immunohistochemistry density analysis quantifies the expression of HMGB1. **d** The levels of colocalization of HMGB1 and OX42 were assessed by using the Pearson coefficient. **e** Representative band of HMGB1 in nuclear and cytosolic fractions using western immunoblot. **f–h** Optical density analysis of the immunoblot bands of HMGB1. β-actin was used as a loading control. **i, j** The levels of acetylation modification of HMGB1. Data are presented as mean ± SEM. *n* = 6, *P < 0.05, *t* test

The increased HMGB1 levels were observed in the CSF of stressed mice (Fig. 1a). Double immunofluorescence analysis for HMGB1 and microglia marker OX42 co-staining showed that HMGB1 was defined to the nuclei of neurons and microglia in the RVLM of control mice (Fig. 1b). However, HMGB1 also translocalized to the cytoplasmic compartment, as represented by increased cytoplasmic HMGB1 immunoreactivities in the RVLM of stressed mice (Fig. 1b–d). In addition, translocation of HMGB1 was further confirmed by western immunoblot analysis of densities using the nuclear and cytosolic fractions of the microglia ex vivo from different groups. The HMGB1 protein from the nuclear fraction was detected as a single band at approximately 28 kDa. In contrast, analysis of the densities of HMGB1 in the cytosolic fraction was detected as two bands exhibited at 28 and 25 kDa, respectively (Fig. 1e). Quantitative analysis of the densities of the HMGB1 bands demonstrated an increase in the amount of HMGB1 in the nucleus; also, there was an increase in HMGB1 expression in the cytosolic fraction after 15-day stress exposure in mice (Fig. 1f–h). Immunoprecipitation (Fig. 1i, j) results showed that HMGB1 acetylation modification was significantly increased, which also suggested the increased cytoplasmic translocation of HMGB1 in stressed microglia.

### siHMGB1 microinjection into the RVLM attenuated M1 activation and lowered BP in SIH mice

Our previous research found that the microglia were activated in the RVLM of stressed rats, while cerebellomedullary cistern infusion minocycline, the M1 activation inhibitor, reduced BP in SIH rats [11]. In this study, we investigated the effect of siHMGB1 on microglia M1 activation ex vivo. The effects of HMGB1 silencing via HMGB1-siRNA transfection into the RVLM were tested using immunoblot, RT-PCR, and HMGB1 immunofluorescent staining of the RVLM tissue, respectively. The data demonstrated that the HMGB1 expression was almost deleted (see Additional file 1).

**Fig. 2:**
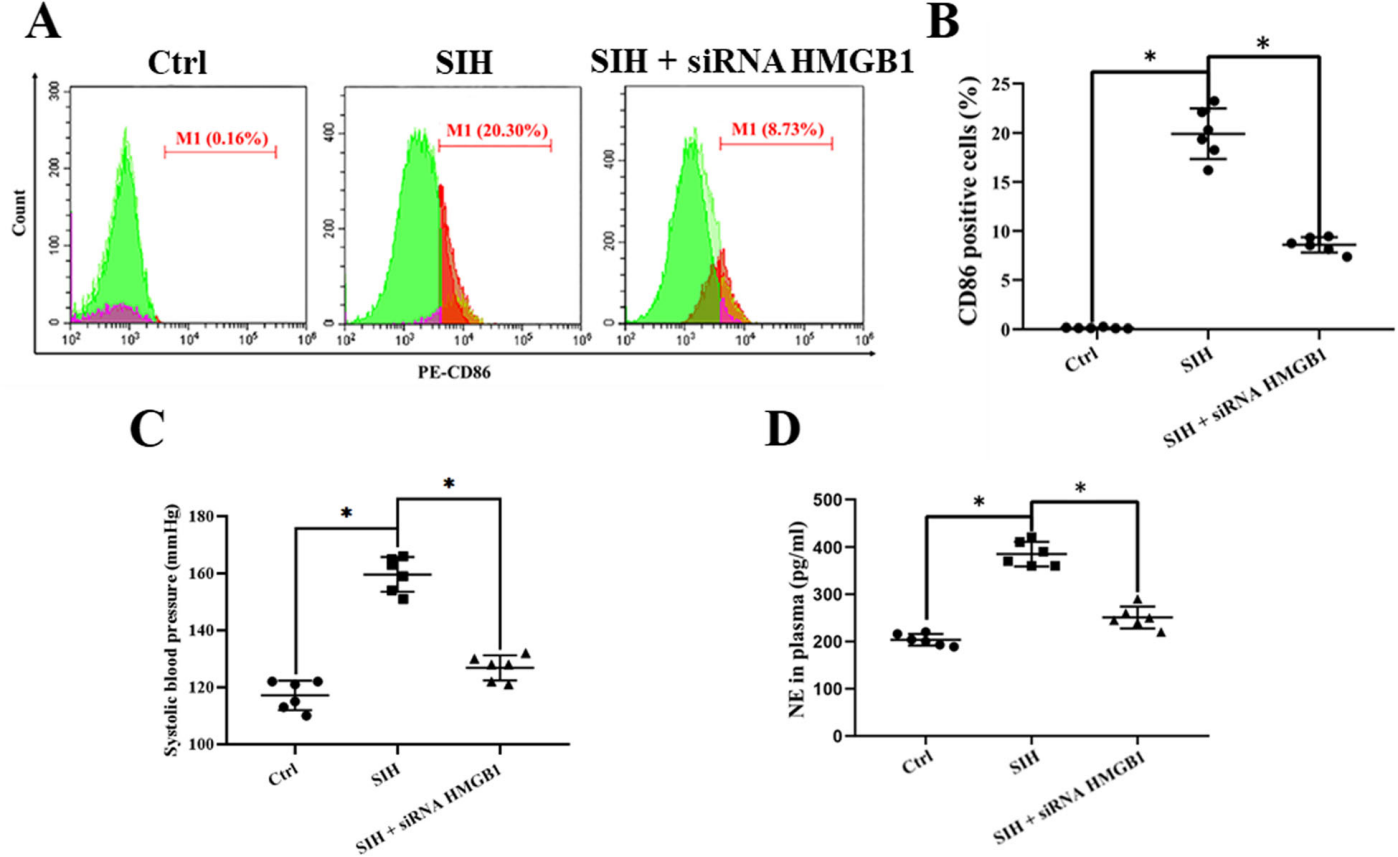
The suppressive effects of siHMGB1 on cardiovascular activity and noradrenaline (NE) levels in mice. **a**, **b** Flow cytometry detection analysis showed the M1 percentage in the RVLM of different groups ex vivo. **c** SBP (mmHg) recording and measurement under conscious conditions in mice. **d** NE plasma concentration in mice. Data are presented as mean ± SEM. n = 6, *P < 0.05, ANOVA LSD test

Flow cytometry detection analysis demonstrated that the percentage of CD86-positive microglia (M1) was increased in the stressed group than that of the siHMGB1-treament microglia (Fig. 2a, b).

Non-invasive tail-cuff BP measurements confirmed there was a significant rise in systolic blood pressure (SBP) of stressed mice compared with that of the control group (159 mmHg ± 8 mmHg vs 115 mmHg ± 7 mmHg, stress vs control), while siHMGB1 microinjection into the RVLM attenuated the rise in BP of stressed mice (128 mmHg ± 6 mmHg vs 159 mmHg ± 8 mmHg, stress+siHMGB1 vs stressed group) (Fig. 2c). Furthermore, siHMGB1 administration reduced the serum norepinephrine (NE) levels; this reflected that peripheral sympathetic activity was reduced in stressed mice (Fig. 2d). The results demonstrated that SBP and NE values in stressed mice were significantly decreased after bilateral injection of the siHMGB1 into the RVLM.

Furthermore, to demonstrate the effects of the siHMGB1 in the RVLM are site specific for cardiovascular responses, microinjections of siHMGB1 were performed 1.0 mm dorsal apart from the RVLM in mice. The changes in SBP and heart rate (HR) were not significant (data not shown).

### Stress-induced RAGE upregulation in microglia, while siHMGB1 microinjection into the RVLM reduced RAGE expression both ex vivo and in vivo

**Fig. 3:**
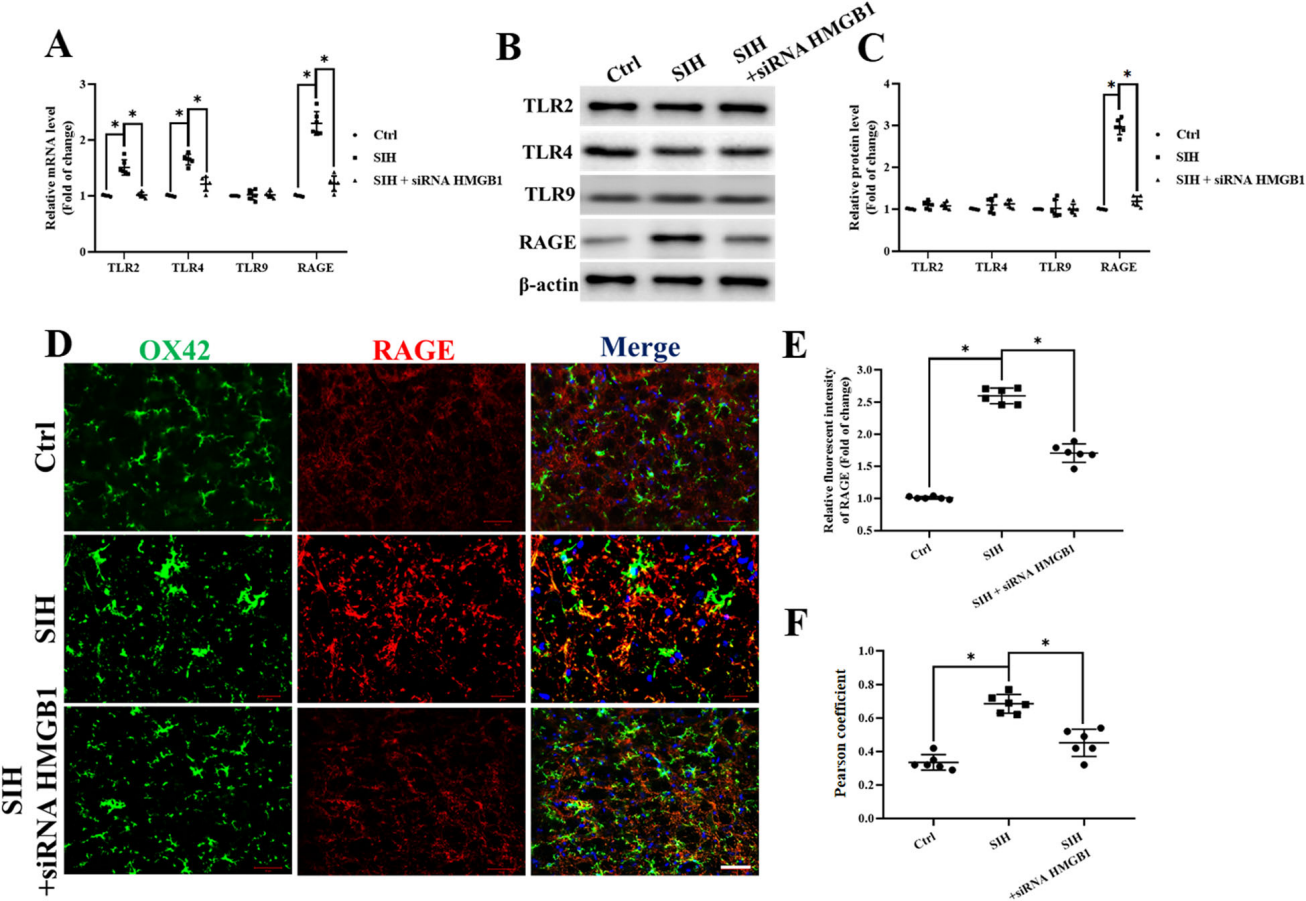
Stress induces RAGE upregulation while HMGB1 silencing in the RVLM reduced RAGE protein expression. **a** TLR2, TRL4, TLR9, and RAGE mRNA expression levels in microglia ex vivo were determined by quantitative real-time PCR. **b**, **c** The protein was obtained and used to measure the differences in the TLR2, TRL4, TLR9, and RAGE expressions. **d** Double immunohistochemical to check RAGE and microglia colocalization in the RVLM from siHMGB1, control, and stressed mice. (scale bar = 50 μm). **e** Densitometric measurement of RAGE immunopositivities. **f** The levels of Co-localization of RAGE and OX42 was assessed by using the Pearson coefficient. Data are presented as mean ± SEM. *n* = 6, *P < 0.05, ANOVA LSD test

We found that stress increased HMGB1 expression in microglia. As HMGB1 acted as an activator of Toll-like receptors (TLR) and receptors for RAGE, we next examined whether stress exposure would induce changes in its receptor of TLR2, TLR4, TLR9, and RAGE expression in microglia ex vivo. After mice were exposed to stress for 15 days, real-time PCR detection revealed that mRNA expression of the TLR2, TLR4, and RAGE were increased, but the expression of the TLR9 mRNA was not changed in microglia (Fig. 3a). Western blot analysis showed the increase in RAGE protein expression, while TLR2, TRL4, and TLR9 protein expression were not changed in microglia (Fig. 3b, c); this result was not exactly consistent with the real-time PCR results. Immunofluorescent studies revealed a high level of RAGE expression in stressed microglia after 15 days of stress exposure, while siHMGB1 microinjection into the RVLM reduced RAGE protein expression ex vivo (Fig. 3d). Immunofluorescent staining of RAGE in the RVLM of mice exhibited the same change in vivo, which demonstrated that siHMGB1 in the RVLM reduced RAGE expression in microglia in stressed mice (Fig. 3e, f).

### Microglia-specific knockout RAGE attenuated M1 polarization and lowered mean arterial blood pressure (MAP) and sympathetic activities which were indicated by reduced in RSNA in vivo

We further identified whether endogenous HMGB1/RAGE axis was involved in the pathogenesis of SIH. The microglia-specific deletion of RAGE in Cre-CX3CR1/RAGE^fl/fl^ mice was tested using immunoblot, RT-PCR, and double immunofluorescent staining of RAGE and microglia in the RVLM tissue, respectively. The data demonstrated that RAGE in microglia was almost deleted (see Additional file 3).

**Fig. 4:**
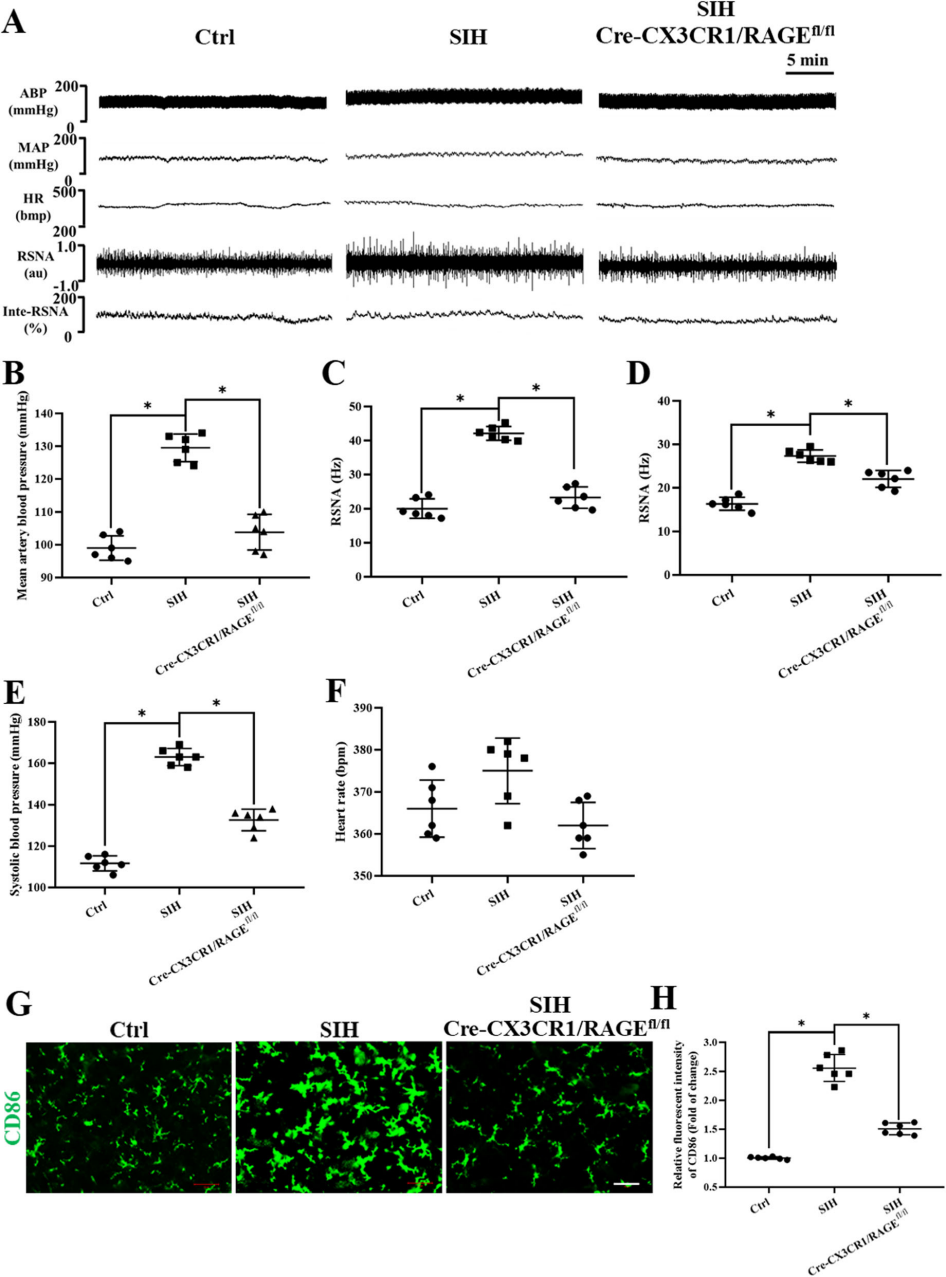
RAGE^−/−^ attenuate the increased M1 polarization, high BP, and great RSNA in stressed mice. **a** Examples of original tracings of RSNA, HR, and BP recording in different groups of mice. Quantitative analysis of **b** MAP, **c**, **d** RSNA, **e** SBP, and **f** heart rate (HR) values for RAGE^−/−^ and stressed mice are shown. **g** Fluorescence images of microglia immunostained for anti-CD86 (M1 marker) in the RVLM from control, stress, and stress + RAGE^−/−^ mice (scale bar = 50 μm). M1 activation was reduced in RAGE^−/−^ mice. **h** Quantitative data on the mean densitometry of CD86 positivities. Data are presented as mean ± SEM. *n* = 6, *P < 0.05, ANOVA LSD test

Changes in RSNA, mean arterial pressure (MAP), and heart rate (HR) in different groups of mice were recorded simultaneously (Fig. 4a). MAP (Fig. 4b) was 105 ± 4 mmHg in RAGE^−/^^−^ plus stressed mice and 129 ± 8 mmHg in stressed mice, which was comparable to MAP values in C57Bl6 wild type mouse (99 ± 5 mmHg) during anesthesia. RSNA was analyzed off-line using both the modified classical discriminator method technique (Fig. 4c) and the wavelet de-noising (Fig. 4d) method. RSNA was lower in control mice compared with that of stressed mice independent of the method used. RSNA was lower in RAGE^−/−^ stressed mice compared to that of SIH mice [discriminator method: stressed 42.4+/− 5.5, stress + RAGE^−/−^ 23.0+/− 4.1 (Fig. 4c), wavelet: spike rate in Hz: stressed 26.5+/− 5.2; stress + RAGE^−/−^ 19.4+/− 3.8 (Fig. 4d)]. The SBP was decreased in RAGE^−/−^ plus stressed mice in comparison with that of stressed mice (Fig. 4e). The HR showed no difference among groups (Fig. 4f). To summarize, MAP and RSNA values decreased significantly in stressed Cre-CX3CR1/RAGE ^fl/fl^ mice, which implied that HMGB1/RAGE axis contributed to augmenting sympathetic activities.

Immunofluorescence staining analysis demonstrated the upregulation positivities of M1 marker CD86 in the RVLM of mice who underwent stress for day 15. And there was morphological change in microglia, compared to that of the control group: microglia in RAGE^−/−^-treated mice were more likely to stay in the ramified form than in the amoeboid form (Fig. 4g, h), which implied that the inflammatory microglia polarization was attenuated by RAGE silencing.

### HMGB1/RAGE axis increased stress-related mitophagy initiation in microglia

**Fig. 5:**
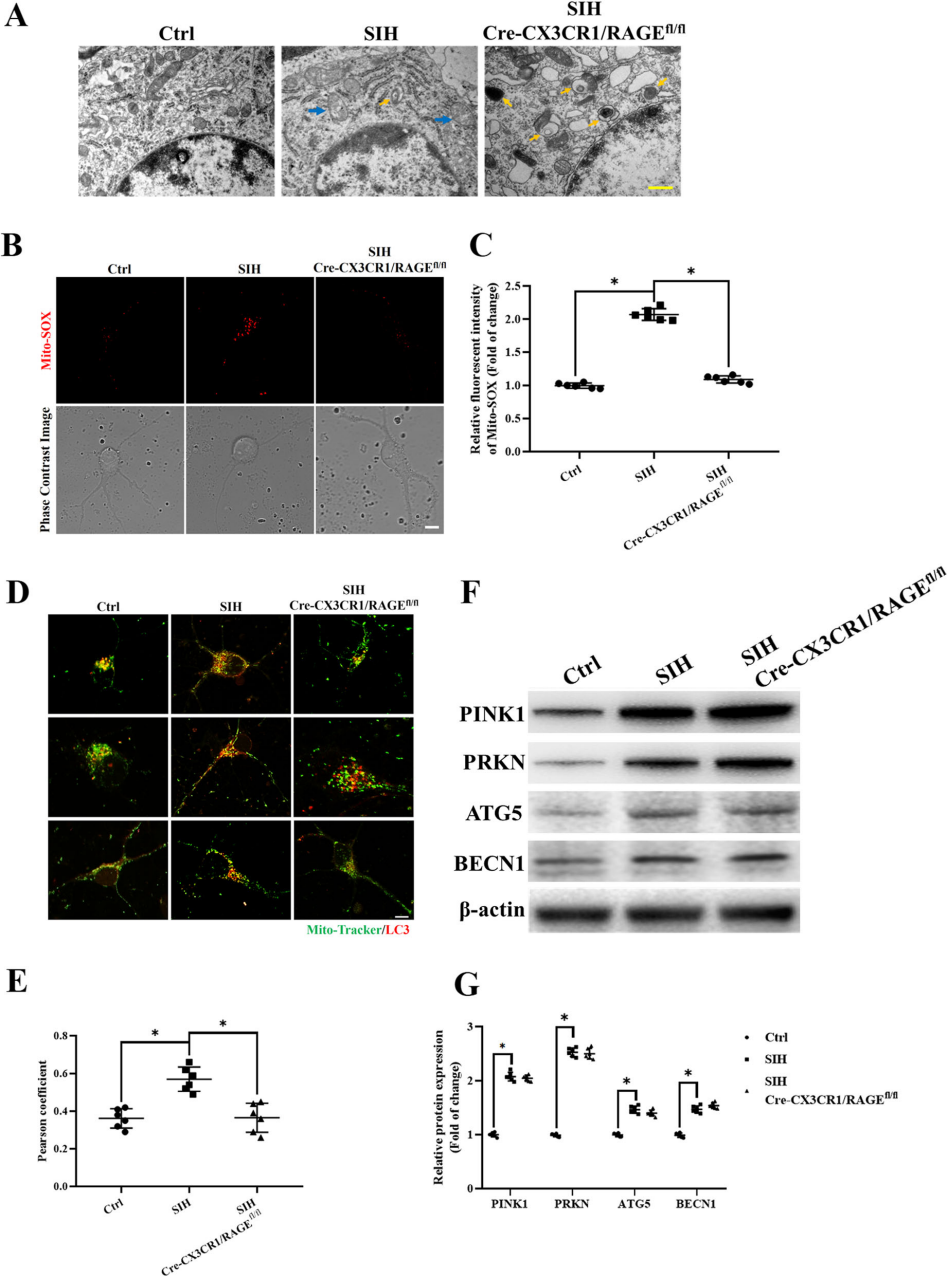
Mitophagy initiation in microglia was increased in the RVLM of stressed mice **a** Representative photomicrographs of microglia from mice preparation for visualization in transmission electron microscopy (60,000×). Blue arrows indicate disruption of the mitochondria with derangement of the mitochondrial crests. Yellow arrows indicate autophagosome. **b**, **c** Representative graph indicates fluorescent probe staining and intensity values of mitoROS; the bottom panel images show the corresponding phase-contrast images (scale bar = 1 μm). **d** Detection of mitochondria (MitoTracker Green) and LC3 (Red) colocalization using confocal microscopy (scale bar = 2 μm). **e** The levels of colocalization of LC-3 and MitoTracker were assessed using the Pearson coefficient. **f**, **g** Representative immunoblot band and densitometry quantitation of autophagy-related protein (PINK1, PRKN, BECN1, and ATG5) expression in microglia in different groups of mice were shown. Data are presented as mean ± SEM. *n* = 6, *P < 0.05, ANOVA LSD test

We have reported that in the stressed rats, the mitochondria were injured and blocked from mitophagy flux in the neurons of the RVLM [11]; however, the mitochondria clearance and autophagy profiles of microglia remain unclear. We found that in most cases, the mitochondria in the stressed microglia showed ultrastructural alterations at the electron microscopy level, for instance, mitochondria with variable size (elongated, fragmental size or swelling) and with reduction or distorted/disrupted cristae (blue arrow heads in Fig. 5a) were found in the stressed microglia. We also found that no typical autophagosomes were observed in the control group (Fig. 5a). However, in stress-treated group, there appeared a marked accumulation of autophagosomes containing mitochondria (yellow arrowheads in Fig. 5a), while RAGE^−/−^ increased the lysosome numbers (Fig. 5a, right panel). The function of the injured mitochondria was then investigated. The mitochondrial ROS production in stressed microglia was increased compared to that of RAGE^−/−^ microglia which returned to near control levels. Panel b shows the mitochondrial ROS production displayed by MitoSOX staining (stress, control and stress + RAGE^−/−^) (Fig. 5b, c).

Dysfunctional mitochondria were recognized and targeted for degradation by mitophagy to maintain mitochondria homeostasis. The existence of mitophagy was then detected by the colocalization of LC3 red with MitoTracker Green. We found that in the stressed microglia, the LC3 red and MitoTracker Green co-staining was increased (Fig. 5d, e), which implied that the increased early-phase mitophagy had occurred.

The abovementioned results suggested that the HMGB1/RAGE axis played a role in initiating early-phase mitophagy. We then measured the early-phase mitophagy protein PINK1-PRKN and autophagosome formation protein BECN1 and ATG5 expression in microglia ex vivo. We found that the PINK1-PRKN, BECN1, and ATG5 protein expression were increased in stressed microglia compared with that of the controls, but their expression did not show significant difference between stress and stress + RAGE^−/−^ group (Fig. 5f, g), which implied that the early-phase mitophagy was not blocked and autophagosome formation was initiated.

### HMGB1/RAGE axis blocked the late stage of mitophagy flux in microglia

The phagosome formation implied the early stage of mitophagy flux was initiated, and the autolysosomal formation represented the late stage of the flux was completed. Since the early phase of mitophagy and autophagosome formation in microglia was initiated by HMGB1/RAGE axis, we then examined the role of HMGB1/RAGE axis in affecting late stage of mitophagy flux of stressed mice. mRFP-GFP-tandem fluorescent LC3 (tf-LC3) virus were transfected into the RVLM to evaluate late stage of autolysosome formation. It is well established that autolysosomes showed GFP fluorescence quenching due to the presence of acidic autolysosomes.

**Fig. 6:**
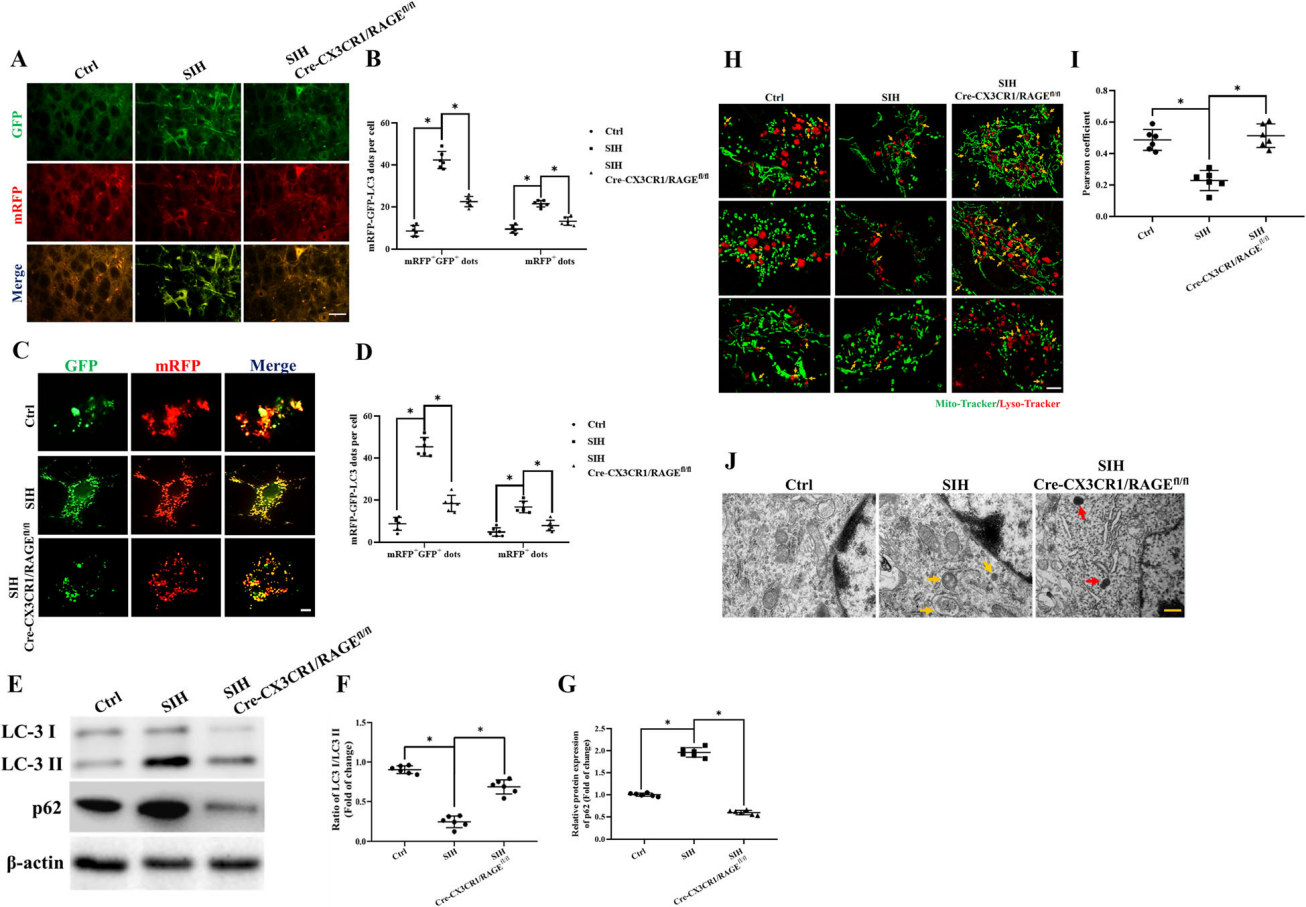
HMGB1/RAGE axis blocks the late stages of mitophagy flux in stressed microglia. LC3 dots were visualized under fluorescent confocal microscope and quantified following mRFP-GFP-tandem fluorescent LC3 adeno-associated virus transfected to the RVLM in vivo (**a**, **b**) (scale bar = 50 μm) and ex vivo (**c**, **d**) (scale bar = 2 μm). **e–g** Whole cell lysates were collected at the indicated time points and analyzed by immunoblotting for LC3II, p62, and β-actin (loading control). The levels of the p62 and LC3-II were increased in the stressed microglia. **h** Microglia stained with Lyso-Tracker-red and MitoTracker Green to label lysosomes and mitochondria colocalization (scale bar = 2 μm). **i** The levels of colocalization of MitoTracker and Lyso-Tracker were assessed by using the Pearson coefficient. **j** Electron micrograph showed few typical autophagosomes in normal cytoplasm of the control microglia. An increased number of mitophagy vesicles were observed in stressed microglia. Red arrows indicate the representative mitophagy, which was visualized as mitochondria-containing autophagosome (scale bar = 0.5 μm). Data are presented as mean ± SEM. *n* = 6, *P < 0.05, ANOVA LSD test

In the RVLM, the late-stage autophagy flux was not impaired by HMGB1/RAGE axis in stress + RAGE^−/−^ mice as revealed by increased GFP fluorescence quenching of GFP-RFP-LC3 puncta in mice (Fig. 6a, b). The decreased mRFP^+^GFP^+^ dots were obtained in microglia ex vivo of stress + RAGE^−/−^ mice, which indicated that activation of HMGB1/RAGE contributed to the autophagic flux blockage in the RVLM microglia of SIH mice (Fig. 6c, d).

During autophagy, a subpopulation of LC3I is converted to LC3II that localizes to autophagosomal membranes. In addition to LC3II/LC3I, p62/SQSTM1 is degraded within the mature autolysosome. The phenomena of LC3II increment is taken as a marker of early-phase mitophagy and p62/SQSTM1 decrement is taken as a marker of late-stage mitophagy flux [33, 34]. Immunoblots showed stressed microglia had higher LC-3 II/I ratios with higher levels of p62. These results indicated that early-stage mitophagy flux is not affected; however, the late stage mitophagy flux is impaired by HMGB1/RAGE axis. In contrast, stress with RAGE^−/−^ treatment resulted in lower LC-3 II/I ratios with an increased degradation of SQSTM1/p62 in stressed microglia (Fig. 6e–g).

Under a microscope, the colocalization of MitoTracker Green with Lysosome-Tracker-red in stressed microglia was decreased in comparison with that of the stressed RAGE^−/−^ group (Fig. 6h, i). Under the transmission electron microscope (TEM), partial autophagic vacuoles contained mitochondrial specific bilayer membrane, cristae, and other structures (yellow arrows), and few lysosomes were observed. The RAGE^−/−^ treatment group showed an increased number of lysosomes (red arrow) (Fig. 6j). These results demonstrated that the HMGB1/RAGE axis impaired the late stage of mitophagy flux in stressed microglia.

### Impaired mitophagy flux in microglia leads to increased NF-κB and inflammatory factor release (neuroinflammation) in vitro

Frank et al. [14] studied the role of the HMGB1 redox state in inflammation and reported that it was disulfide HMGB1 (ds-HMGB1) instead of fully reduced HMGB1 (fr-HMGB1) contributed to the inflammatory responses. We next investigated the effects of exogenous ds-HMGB1 on NF-κB activation and PIC release in vitro. The microglia were treated with ds-HMGB1(1000 ng/ml) [14] for 4 h. Furthermore, the autophagy inducer (1 nM rapamycin) [35] and/or lysosomal inhibitor (5 μM chloroquine) [36] was used to explore the relationship between the autophagy and inflammation.

**Fig. 7:**
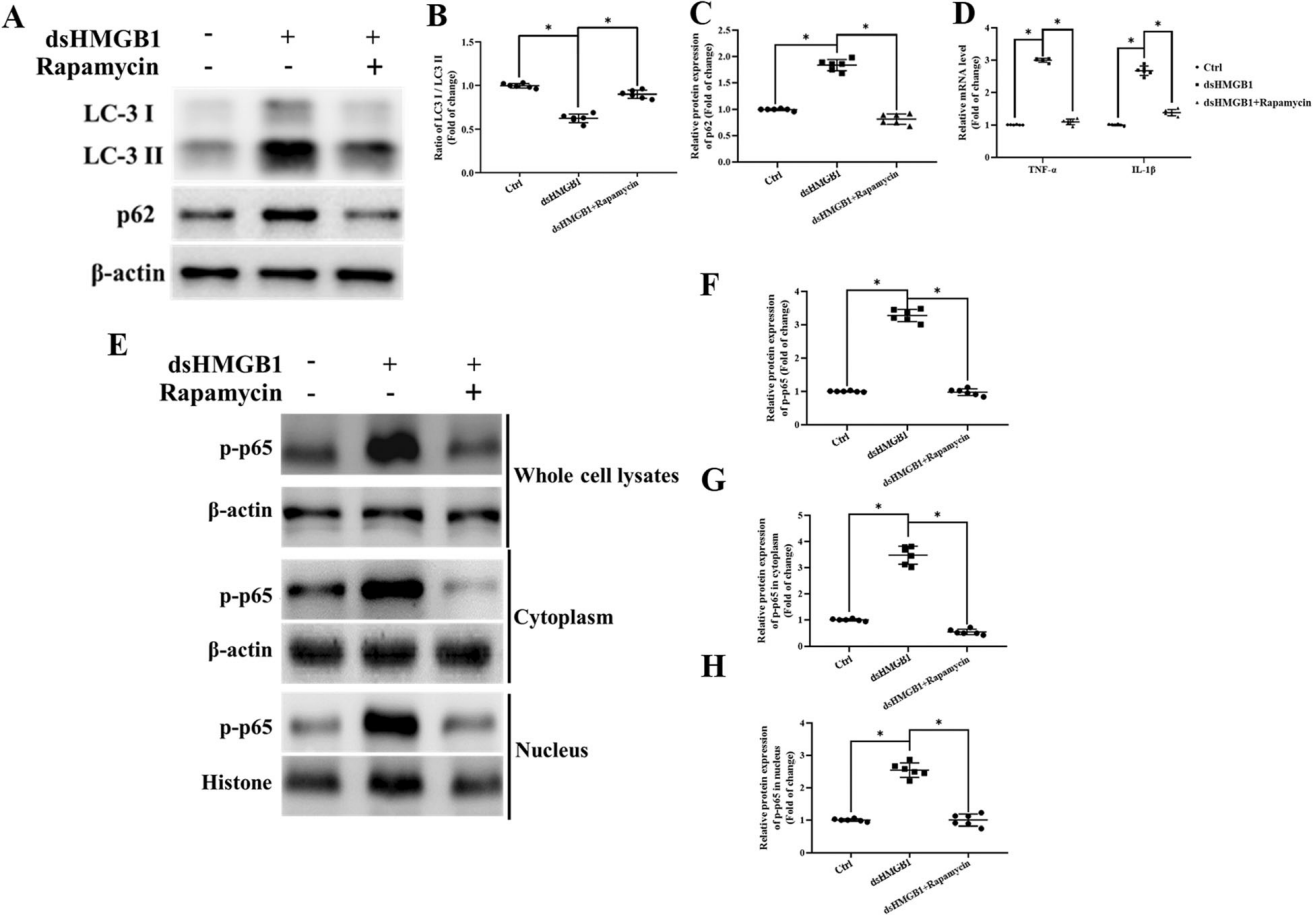
Ds-HMGB1 induced increased NF-κB activation resulted from disruption of microglial autophagy. **a** Representative immunoblot bands of LC3 and SQSTM1/p62 in different groups of microglia. **b**, **c** Optical density analysis of LC3 and SQSTM1/p62 immunoblot bands. **d** the mRNA expression of IL-1β and TNF-α was increased in ds-HMGB1-treated microglia in vitro. **e** NF-κB p65 was detected from nuclear and cytosolic fractions extracted from microglia using western immunoblot. **f–h** Optical density analysis of phosphorylation of NF-κB p65 bands both in nuclear and cytosolic fractions of microglia. β-actin and histone were used as the cytoplasmic or nuclear loading control, respectively. Data are presented as mean ± SEM. *n* = 6, *P < 0.05, ANOVA LSD test

As presented in Fig. 7a–c, ds-HMGB1 treatment resulted in a decreased degradation of SQSTM1/p62 while cotreatment with rapamycin resulted in higher LC-3 II/I ratios with lower levels of p62 in microglia which indicated that mitophagy flux is facilitated by rapamycin. Figure 7d shows that compared with that of the control group, the mRNA expression of IL-1β and TNF-α were increased in ds-HMGB1 treatment microglia. NF-κB activation was assessed by measuring its phosphorylation level and its nuclear translocation. NF-κB p65 were detected both in nuclear and cytosolic fractions extracted from microglia. The release of PICs, p-p65 expression, and its nuclear translocation was significantly decreased in ds-HMGB1 cotreatment with the rapamycin group in comparison with that of the rapamycin absence group (Fig. 7e–h). We demonstrated that facilitation of the mitophagy flux would attenuate proinflammatory state in microglia.

### HMGB1/RAGE axis mediated autophagy-related lysosomal dysfunction in microglia

**Fig. 8:**
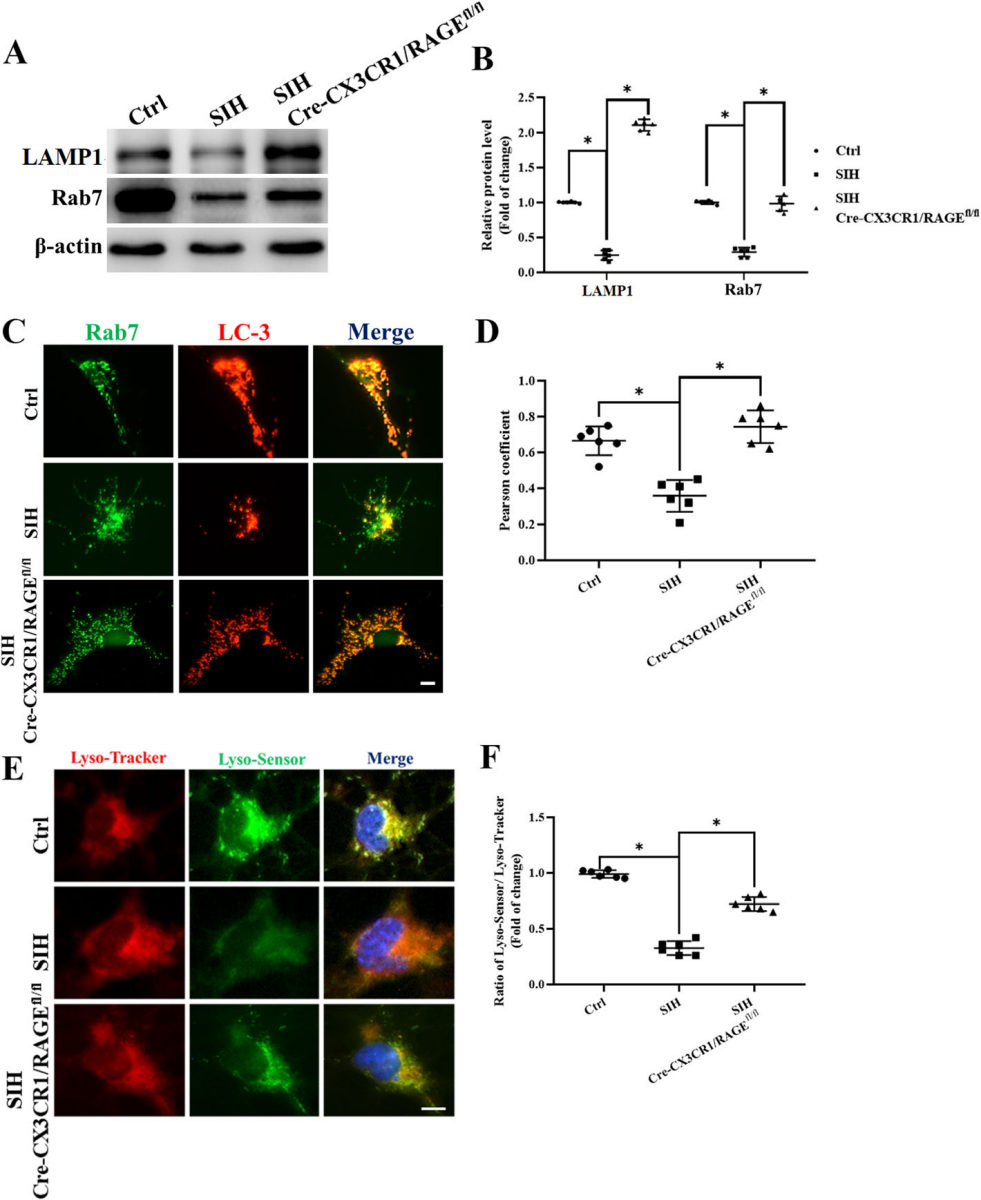
Lysosomal function is impaired in RAGE^−/−^ mice. **a** Representative western blot images and **b** statistical analysis of RAB7 and LAMP1 expression in microglia. **c** Double immunofluorescence showed colocalization of RAB7 and LC3 which was analyzed by confocal fluorescence microscopy. **d** The level of colocalization of LC3 (red) and RAB7 (green) was assessed by using the Pearson coefficient (scale bar = 2 μm). **e**, **f** Acidation of lysosome was detected by using the Lyso-Tracker/Lyso-Sensor staining (scale bar = 2 μm). Data are presented as mean ± SEM. *n* = 6, *P < 0.05, ANOVA LSD test

**Fig. 9:**
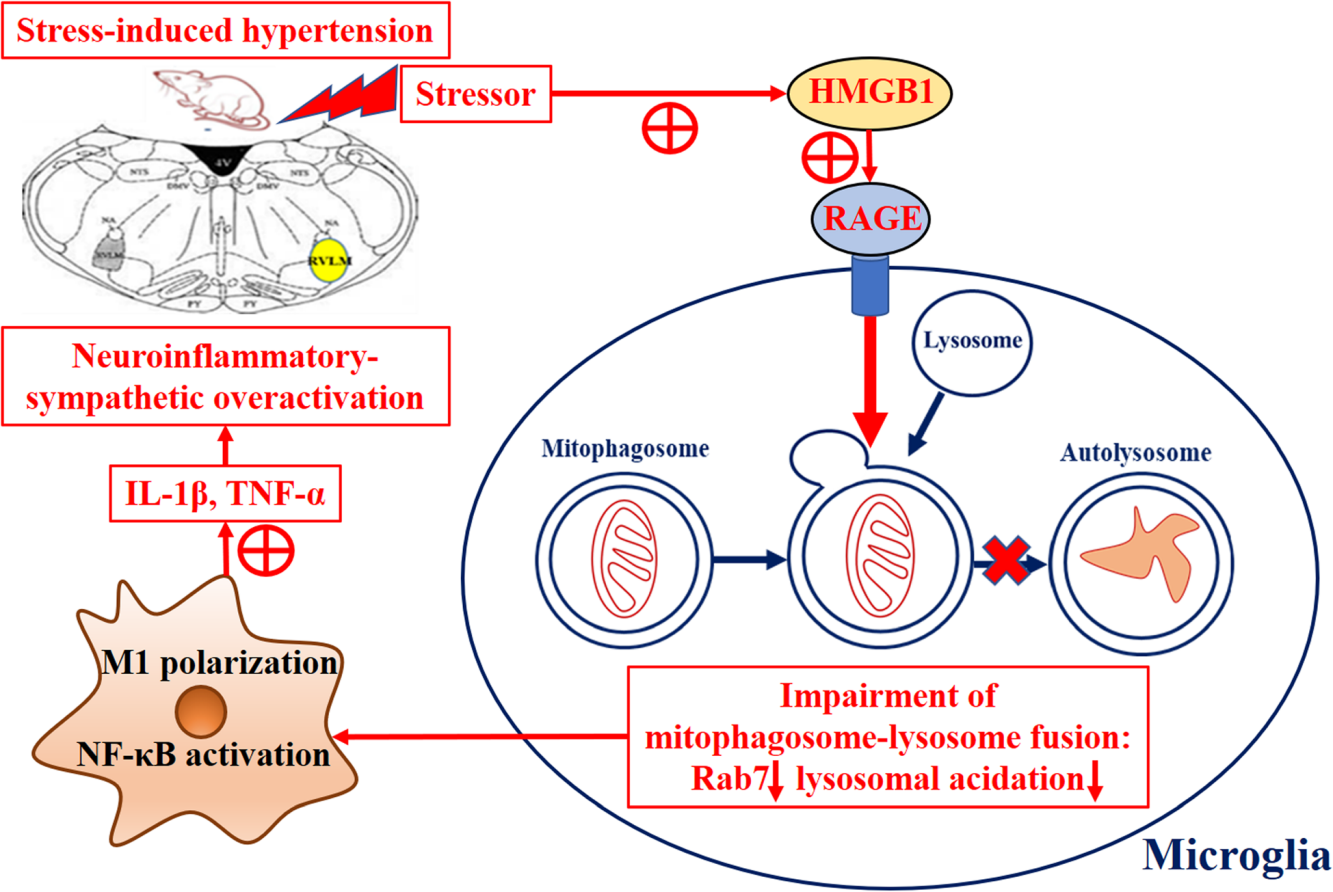
Schematic diagram displaying HMGB1/RAGE axis mediating stress-induced RVLM neuroinflammation via impairing mitophagy flux in microglia

Considering that the capacity of lysosomal degradation is a rate-limiting factor for autophagic flux, we next assessed the function of lysosomes. The activation of the roles and mechanisms of Rab7 in endosomal and endolysosomal transport were greatly reported [37, 38]. We then assessed the expression of RAB7 and lysosome-associated membrane proteins 1 (LAMP1) (Fig. 8a) in microglia. Furthermore, we evaluated the acidation of lysosome by using the Lyso-Tracker/Lyso-Sensor staining. The results showed that the expression of RAB7 and LAMP1 were decreased in the stressed microglia in comparison with that of RAGE^−/−^ microglia (Fig. 8b). The colocalization of LC3 and RAB7 were significantly decreased (Fig. 8c, d), concomitant with lower 488/590 ratio, implicating that stress microglia possessed less acidic lysosomal pH. In contradiction, RAGE^−/−^ in stressed microglia reversed this effect indicated by significantly elevated acidifying lysosomes in comparison with that of the stressed group (Fig. 8e, f). Taken together, these results revealed that the impairment of autophagic flux in stressed microglia is mediated by the HMGB1/RAGE axis, which is closely associated with the reduced degradation capability of autolysosomes and lysosomes (Fig. 9).

## Discussion

As a worldwide and high morbidity disorder, unraveling the causal mechanisms of hypertension remains a challenge [39]. With the advancement of the research on the neurogenic pathogenesis of hypertension, it has been discovered that the pathogenesis of hypertension is closely related to environmental stress [40], mental tension, sympathetic excitability, neurological dysfunction, and other mechanisms [41, 42]. In our present study, we evidenced increased occurrence of microglial alteration in the RVLM because of chronic stress exposure in mice as shown by an activated and deramified morphology and increased activity markers of microglia, while it was attenuated in HMGB1/RAGE ablated mice. In addition, a decreased M1 polarization in isolated microglia generated from HMGB1-deficient and RAGE^−/−^ mice was noticed. Further, we assessed mitophagy, one of the major mechanisms in the RVLM of brain that keeps injured mitochondria clearance to maintain mitochondrial homeostasis. Indeed, microglial inflammation was conditioned upon defective microglial autophagy in stressed mice. Facilitating autophagy by treatment with rapamycin was sufficient to decrease NF-κB activation and PIC release in cultured primary microglia. Using genetic method to interfere the major receptors of HMGB1 expressed in the microglia, we identified RAGE, one of HMGB1 receptors, as the mediator of both impairing autophagy and leading to proinflammatory effects. In line with these results, the addition of exogenous ds-HMGB1 stimulated primary microglial cells showed similar proinflammatory effects. This study confirmed the hypothesis that HMGB1/RAGE axis impairs the late stage stress-mediated mitophagy flux and thereby promoting the M1 polarization and PIC release in the RVLM, which subsequently elicit neuroinflammation-sympathetic activation effect in SIH. Microglia inflammation is thought to play a role in the pathogenesis of the hypertension. Evidences implied that the neuroinflammatory cascades were involved in alterations of cross-talks between glial cells and neurons as a consequence of the activation of microglia and astrocytes. Therefore, minocycline or only microglia deletion was demonstrated to be effective to decrease blood pressure. However, such manipulation did not completely reverse blood pressure to a normal level. We are highlighting the importance of mitophagy flux not only in neurons, but also in microglia, in causing neuroinflammation in stress-induced hypertension mice.

Stress can be perceived by the brain and induces a series of neuroendocrine responses in either a rapid or long-term manner. SIH model has great similarity with human essential hypertension, which is of great value in understanding the pathogenesis and risk factors of hypertension [43–45]. Evidences show psychological and physical stress can either enhance or suppress immune responses depending on a variety of factors such as duration and severity of stressful situation. Mitophagy flux involves the activation, inflammatory response, and survival of microglia [46, 47]; however, the detailed mechanisms remain largely unknown.

Few literatures reported that independent noise stressor can induce hypertension so far. Epidemiological evidences show that persistent chronic noise exposure increases the risk of cardiometabolic diseases, including arterial hypertension, coronary artery disease, diabetes mellitus type 2, and stroke [66]. Du et al. [48] showed that microglial cells to infrasound with a main frequency of 16 Hz and a sound pressure level of 130 dB for 2 h resulted in a significant activation of microglia cells and upregulated their expression of corticotrophin-releasing hormone (CRH)-R1 in the hypothalamic paraventricular nucleus (PVN) in vivo. Their in vitro data further revealed that infrasound directly induced microglial activation and upregulated their CRH-R1 expression, which suggests that in addition to the neurons, microglial cells are the effector cells for the infrasound-induced stress as well.

Dong et al. [49] and Wang et al. [50] reported that the 2-week foot shock treatment significantly increased SBP. Our modified method used a series of foot electric shocks synchronous with noise stimulation for 15 days to create a model of stress-related hypertension [24]. On the one hand, noise is a specific stress that activates the autonomic nervous system and endocrine signaling. At around day 7, when the conditioned reflex (one noise of buzzer bell represents the arrival of an electric shock) was developed, the electric shock was removed irregularly to reduce its potential physical damage to the mice. Given the above experimental model, we cannot exactly differentiate the effects of noise from electric stroke stressor on autophagy reflux. In different animal disease models and pathophysiological settings, the autophagic reflux occurred from 24 h to several weeks [51, 52]. In our present study, we found that after the 7th day, the tail artery SBP of the SIH rats kept rising gradually around the 15th day. Interestingly, the autophagy flux impairment and the microglia activation were constantly observed from 7th day to the 15th day until we sacrificed the mice.

In other settings, the impaired autophagic function results from a decline of autophagy genes including ATG-5, ATG-7, and BECN-1 or increased MTORC1 activity in human and/or rodent brain with aging or aging-related disease [53–56]. Future studies need to be performed for better understanding of molecular and cellular mechanisms on how autophagy in different stages or conditions regulates the activation and inflammatory response in microglia under SIH conditions.

Recent evidence suggested that high-mobility group box 1 (HMGB1) may also mediate stress-induced sensitization of neuroinflammatory responses [57]. HMGB1 has two DNA-binding regions (A box and B box) [58]. The cytokine active region gene of HMGB1 is mainly located in B box, among which the initial 40 peptides can induce the production of cytokines such as TNF-α and IL-6, which is a highly conserved functional domain of proinflammatory response [13, 59]. The regulation of autophagy and apoptosis by HMGB1 is related to its own redox state [60]. Thus, in our experiment, we used ds-HMGB1 to stimulate the primary microglia.

The inflammation and autophagy interacted in the form of bidirectional causality. Inflammation mediators can disrupt the clearance of misfolded proteins. To the contrary, the impaired autophagy causes inflammation [46]. We found that the lysosomal functional protein, represented by the expression of RAB7 and LAMP1 were decreased in the stressed microglia, while RAGE^−/−^ in microglia reversed this effect and increased the acidity of lysosome. How chronic mild stress causes the lysosomal dysfunction need to be further investigated. When mitophagy flux was facilitated using rapamycin, which was the autophagy inducer, this results in attenuated NF-κB activation in vitro. We found that stressed microglia showed increased mitochondria ROS (mitoROS). The mitochondrion and mitochondrially produced ROS are essential for autophagy and mitophagy [61]. Oxidative stress might cause redox modification of autophagy proteins and impact of autophagy deficits, such as TFEB and lysosomal-related protein [62, 63]. In addition, we found that exogenous HMGB1 reduced the ATP production and mitochondria respiration, while rapamycin can reverse these energy problems, while chloroquine, an inhibitor of lysosomes and autophagic protein degradation, abrogated these beneficial effects (Additional file 5). These data implied the mitophagy impairment caused the mitochondria dysfunction, which might relate to lysosomal dysfunction in dsHMGB1 stimulated microglia.

Our results suggest that the impaired mitophagy flux might initiate the inflammatory state in the RVLM of stress mice. It is well defined that misfolded protein accumulation and aberrant level of inflammatory mediators in the brain can eventually trigger sympathetic nervous activity [64–66]. We found that HMGB1/RAGE axis impaired mitophagy flux and subsequently leading to increased release of proinflammatory mediators at a molecular level. The immediate early gene c-fos expression was increased in the RVLM neurons of SIH mice in comparison with that of Cre-CX3CR1/RAGE ^fl/fl^ stressed mice (see Additional file 6). We suggested that the impaired neuron mitophagy might excite the neurons partially via releasing excitatory neurotransmitters [11], while our present study implied that the impaired mitophagy flux in microglia might cause neuroinflammation, which in turn exaggerate the excitation of presympathetic neurons in the cardiovascular center of the RVLM.

Lastly, the combination of electrical stimulation and noise causes the rapid increase of blood pressure of murine in 2 weeks of stress, and the maintenance time can be up to 3 weeks. It has been reported that long-term cold stimulation (4–5 °C) for 5 weeks caused significant increase of blood pressure in mice, and combined with high-salt diet stimulation, cold stimulation caused hypertension more significantly. Both stress models are reversible hypertension due to the reversibility of elevated BP and the absence of morphological and pathological changes in the heart and vessels [50]. In this setting, if the causal relationship of impairing stress-induced mitophagy flux resulting in neuroinflammation is proven correct, then via direct or indirect manipulations of signaling pathways of mitophagy flux, it could theoretically reduce neuroinflammation and SANS activity in pre-hypertension individuals. Further investigations on the immune reactivity in hypertension may result in the identification of new strategies for the treatment of the hypertension.

## Conclusion

Collectively, we demonstrated that the HMGB1/RAGE axis impaired the stress-induced mitophagy flux in microglia, thereby giving rise to microglia-mediated neuroinflammation in the RVLM. Subsequently, microglia-mediated neuroinflammation will increase sympathetic vasoconstriction drive in the RVLM. Inhibition of the HMGB1/RAGE axis reduced the sympathetic vasoconstriction drive in the RVLM (see figures of the content). This study provided a new strategy and target for interfering SIH.

## Supplementary information

Additional file 1: **Figure S1.** Microinjection HMGB1 siRNA into RVLM to silence HMGB1 in mice. (A-C) Immunofluorescent staining showed the expression of HMGB1 in microglia of RVLM in mice. The level of co-localization of RAGE and OX42 was assessed by using the Pearson coefficient. (Scale bar = 50 μm) (D-E) Western blot results showed that Deleted-HMGB1 protein was detected in HMGB1 silencing mice via siHMGB1 microinjection into RVLM of mice. (F). RT-PCR result showed that HMGB1 mRNA in RVLM have been deleted in siHMGB1 microinjection mice. Data are presented as mean ± SEM. *n* = 6, *P < 0.05, t test.

Additional file 2: **Figure S2.** Identification of the RVLM microinjection sites. The microinjection site of RVLM was stained by neutral red. Panel left represented the schematic graphs and panel right showed photomicrograph taken, respectively. The black arrow indicated the microinjection sites of RVLM.

Additional file 3: **Figure S3.** The mRNA and protein of RAGE have been depleted in microglia of RVLM in Cre-CX3CR1/RAGE^fl/fl^ mice. (A-C) Immunofluorescent staining showed the expression of RAGE in microglia of RVLM in mice. The level of co-localization of RAGE and OX42 was assessed by using the Pearson coefficient. (Scale bar = 50 μm) (D-E) Western blot results showed that RAGE protein in RVLM have been deleted in Cre-CX3CR1/RAGE ^fl/fl^ mice. (F) RT-PCR result showed that RAGE mRNA in RVLM have been deleted in Cre-CX3CR1/RAGE ^fl/fl^ mice. Data are presented as mean ± SEM. n = 6, *P < 0.05, t test.

Additional file 4: **Figure S4.** Purity identification of microglia isolation and culture. Cultured microglia cells were identified by microglial marker anti-OX42 (CD11b /c) staining. The results showed that the purity of microglia cells cultured was more than 95%. (Scale bar = 10 μm).

Additional file 5: **Figure S5.** Mitochondrial respiratory function measurement by Seahorse cell metabolometer. The effects of dsHMGB1 and dsHMGB1 co-treatment with rapamycin/chloroquine on mitochondrial aerobic respiration of microglia were detected by Seahorse cell metabolometer. The results showed that dsHMGB1 reduced MG mitochondrial basal respiration, ATP synthesis, and decreased maximal respiration and respiratory potential. Induction of autophagy improved mitochondrial respiration function. Data are presented as mean ± SEM. n = 6, *P < 0.05, ANOVA LSD test.

Additional file 6: **Figure S6.** Targeting on RVLM microglia-specific RAGE deletion inhibited presympathetic neurons excitation in stressed mice. (A) The immunofluorescent staining showed colocalization of the immediate early gene c-fos (red) with neural marker PGP9.5 (green), c-fos protein expressed in the nuclear of the neurons. (Scale bar = 100 μm) (B) c-fos expression was increased in RVLM neurons of SIH mice in comparison with that of Cre-CX3CR1/RAGE ^fl/fl^ stressed mice. Data are presented as mean ± SEM. n = 6, *P < 0.05, ANOVA LSD test.

## Abbreviations

Ang II: Angiotensin II
BP: Blood pressure
CNS: Central nervous system
CQ: Chloroquine
ds-HMGB1: Disulfide HMGB1
ELISA: Enzyme-linked immunosorbent assay
HMGB1: High-mobility group box 1
HR: Heart rate
IL-1β: Interleukin-1β
LAMP1: Lysosomal-associated membrane proteins 1
LC3: Microtubule-associated protein light chain 3
MAP: Mean arterial pressure
MG: Microglia
mitoROS: Mitochondrial Reactive Oxygen Species
mTOR: Mammalian target of rapamycin
NF-κB: Nuclear factor-kappa B
NLRP3: The nucleotide-binding oligomerization domain (NOD)-like receptor containing pyrin domain 3
p62: SQSTM1 (sequestosome 1)
PICs: Proinflammatory cytokines
PVN: Hypothalamic paraventricular nucleus
RAGE: Advanced glycation end products receptor
Rap: Rapamycin
RAS: Renin-angiotensin system
ROS: Reactive oxygen species
RSNA: Renal sympathetic nerve activity
RVLM: Rostral ventrolateral medulla
SBP: Systolic blood pressure
SDS-PAGE: Sodium dodecyl sulfate-polyacrylamide gel electrophoresis
SIH: Stress-induced hypertension
SNA: Sympathetic nerve activity
SNS: Sympathetic nervous system
TNF-α: Tumor necrosis factor α
WT: Wild type
