# Supplementary figures and images for: HMGB1/RAGE axis mediates stress-induced RVLM neuroinflammation in mice via impairing mitophagy flux in microglia
Source: J Neuroinflammation. 2020 Jan 10;17:15. doi: 10.1186/s12974-019-1673-3 (PMC6953162; doi:10.1186/s12974-019-1673-3)

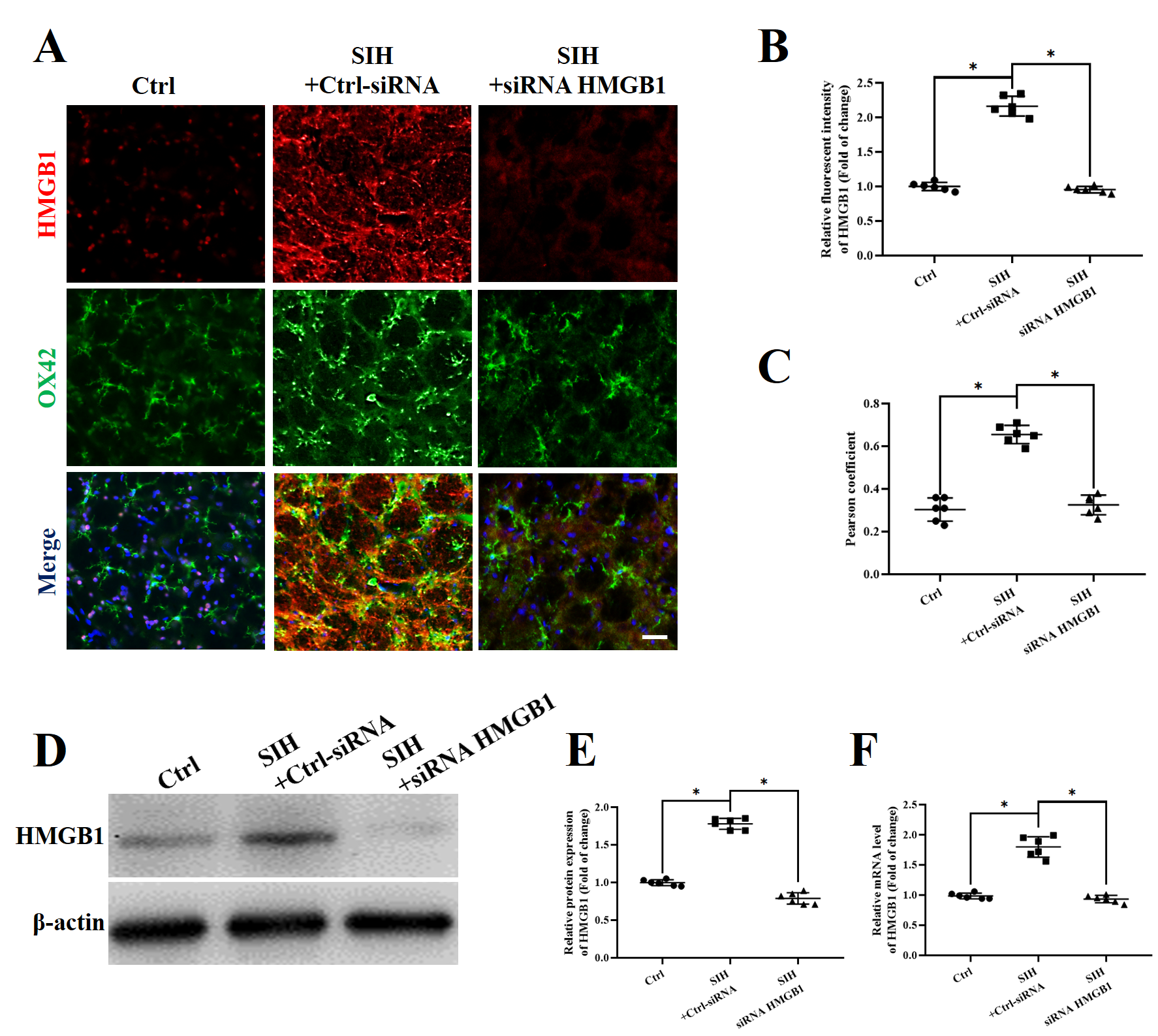

Supplement: Supplementary file 1 — Figure S1. Microinjection HMGB1 siRNA into RVLM to silence HMGB1 in mice. (A-C) Immunofluorescent staining showed the expression of HMGB1 in microglia of RVLM in mice. The level of co-localization of RAGE and OX42 was assessed by using the Pearson coefficient. (Scale bar = 50 μm) (D-E) Western blot results showed that Deleted-HMGB1 protein was detected in HMGB1 silencing mice via siHMGB1 microinjection into RVLM of mice. (F). RT-PCR result showed that HMGB1 mRNA in RVLM have been deleted in siHMGB1 microinjection mice. Data are presented as mean ± SEM. n = 6, *P < 0.05, t test. [file 12974_2019_1673_MOESM1_ESM.bmp]

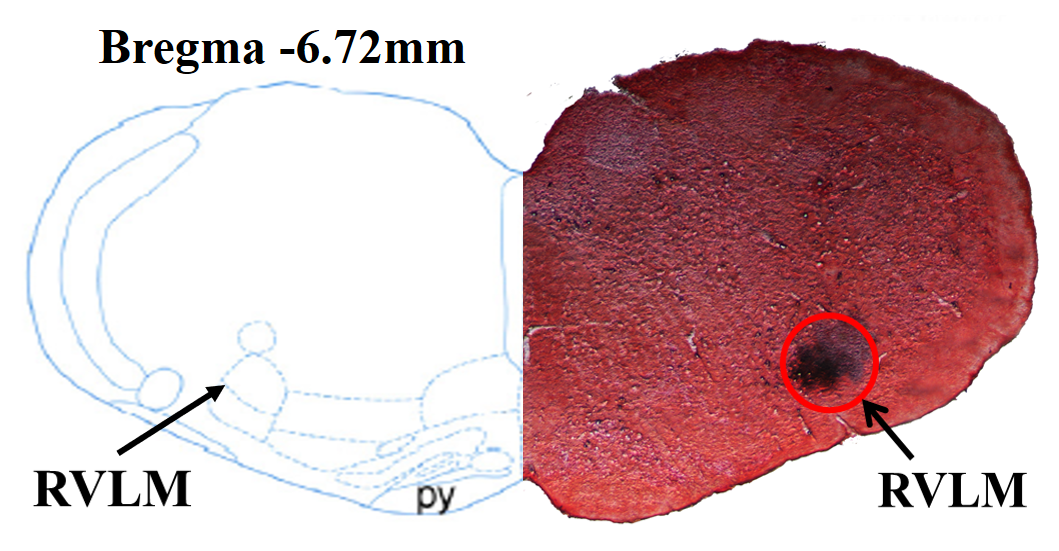

Supplement: Supplementary file 2 — Figure S2. Identification of the RVLM microinjection sites. The microinjection site of RVLM was stained by neutral red. Panel left represented the schematic graphs and panel right showed photomicrograph taken, respectively. The black arrow indicated the microinjection sites of RVLM. [file 12974_2019_1673_MOESM2_ESM.bmp]

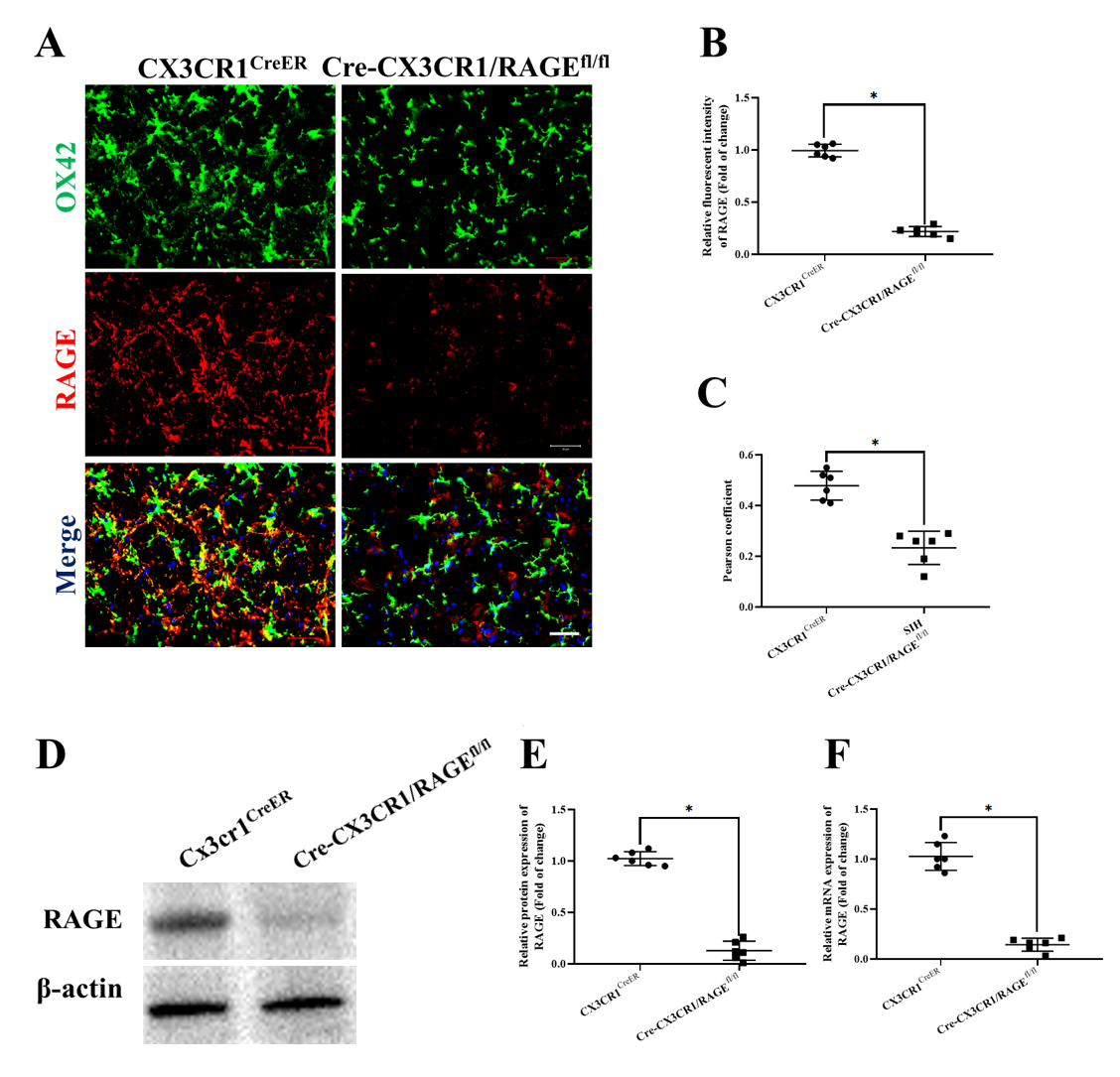

Supplement: Supplementary file 3 — Figure S3. The mRNA and protein of RAGE have been depleted in microglia of RVLM in Cre-CX3CR1/RAGEfl/fl mice. (A-C) Immunofluorescent staining showed the expression of RAGE in microglia of RVLM in mice. The level of co-localization of RAGE and OX42 was assessed by using the Pearson coefficient. (Scale bar = 50 μm) (D-E) Western blot results showed that RAGE protein in RVLM have been deleted in Cre-CX3CR1/RAGE fl/fl mice. (F) RT-PCR result showed that RAGE mRNA in RVLM have been deleted in Cre-CX3CR1/RAGE fl/fl mice. Data are presented as mean ± SEM. n = 6, *P < 0.05, t test. [file 12974_2019_1673_MOESM3_ESM.bmp]

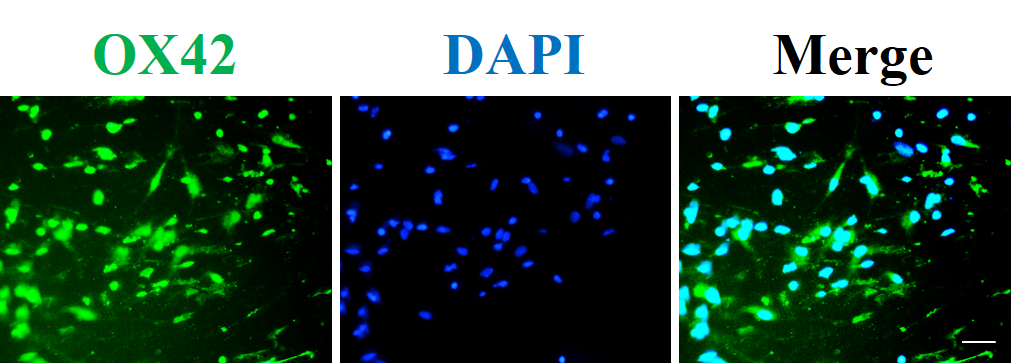

Supplement: Supplementary file 4 — Figure S4. Purity identification of microglia isolation and culture. Cultured microglia cells were identified by microglial marker anti-OX42 (CD11b /c) staining. The results showed that the purity of microglia cells cultured was more than 95%. (Scale bar = 10 μm). [file 12974_2019_1673_MOESM4_ESM.bmp]

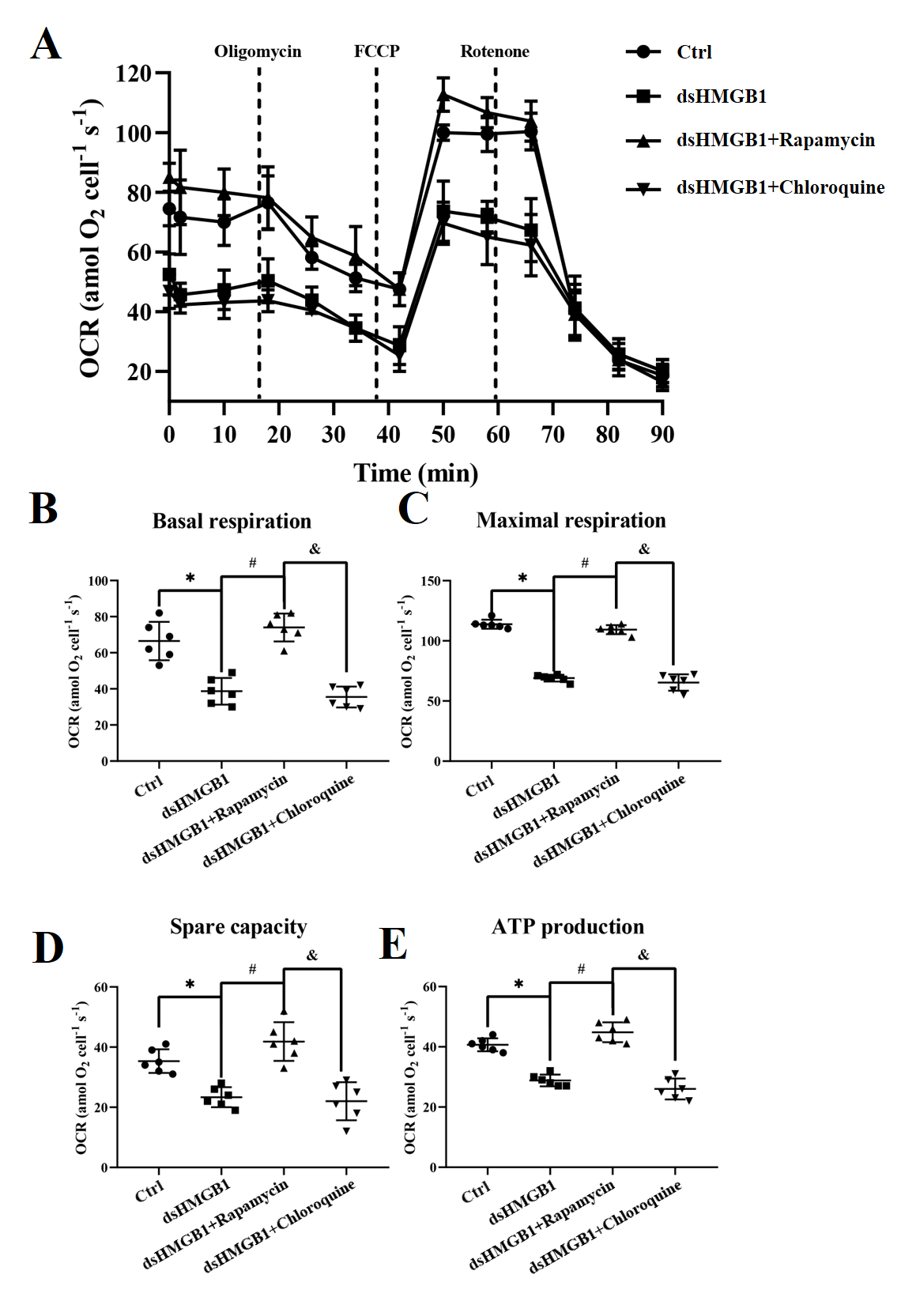

Supplement: Supplementary file 5 — Figure S5. Mitochondrial respiratory function measurement by Seahorse cell metabolometer. The effects of dsHMGB1 and dsHMGB1 co-treatment with rapamycin/chloroquine on mitochondrial aerobic respiration of microglia were detected by Seahorse cell metabolometer. The results showed that dsHMGB1 reduced MG mitochondrial basal respiration, ATP synthesis, and decreased maximal respiration and respiratory potential. Induction of autophagy improved mitochondrial respiration function. Data are presented as mean ± SEM. n = 6, *P < 0.05, ANOVA LSD test. [file 12974_2019_1673_MOESM5_ESM.bmp]

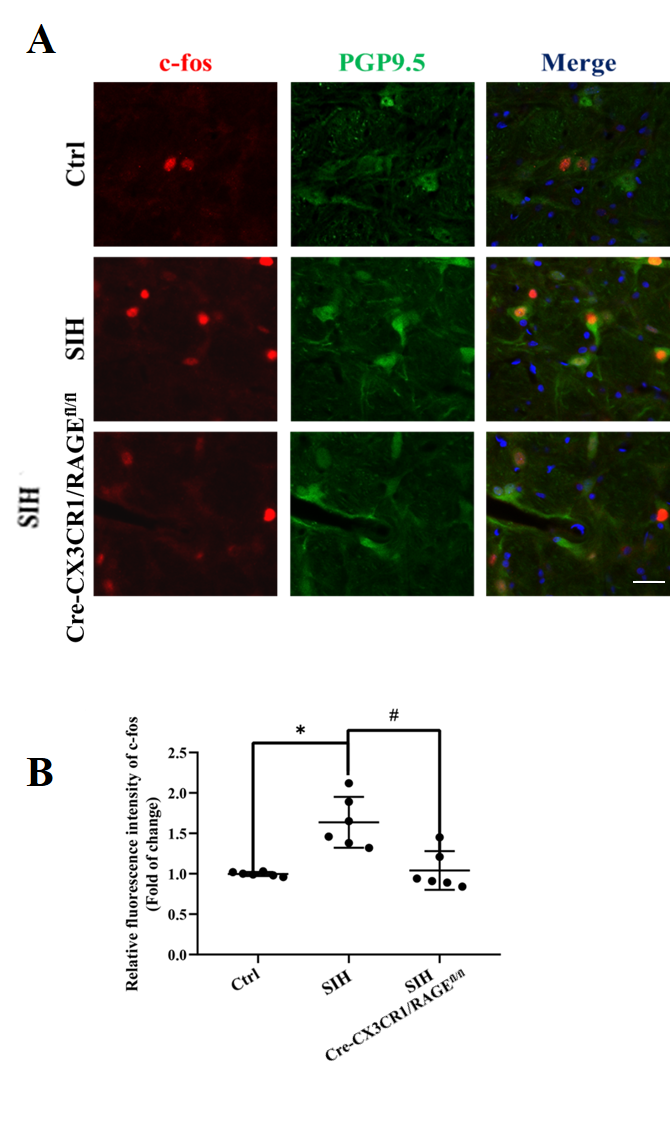

Supplement: Supplementary file 6 — Figure S6. Targeting on RVLM microglia-specific RAGE deletion inhibited presympathetic neurons excitation in stressed mice. (A) The immunofluorescent staining showed colocalization of the immediate early gene c-fos (red) with neural marker PGP9.5 (green), c-fos protein expressed in the nuclear of the neurons. (Scale bar = 100 μm) (B) c-fos expression was increased in RVLM neurons of SIH mice in comparison with that of Cre-CX3CR1/RAGE fl/fl stressed mice. Data are presented as mean ± SEM. n = 6, *P < 0.05, ANOVA LSD test. [file 12974_2019_1673_MOESM6_ESM.bmp]
